# ﻿Four novel endolichenic fungi from *Usnea* spp. (Lecanorales, Parmeliaceae) in Yunnan and Guizhou, China: Taxonomic description and preliminary assessment of bioactive potentials

**DOI:** 10.3897/mycokeys.118.155248

**Published:** 2025-06-02

**Authors:** Runlei Chang, Zhaoqi Yan, Jibo Jiang, Yichen Wang, Hongli Si, Tanay Bose, Congcong Miao

**Affiliations:** 1 College of Life Science, Shandong Normal University, Jinan, China Shandong Normal University Jinan China; 2 Dongying Institute, Shandong Normal University, Dongying, China Shandong Normal University Dongying China; 3 Department of Biochemistry, Genetics & Microbiology, Forestry and Agricultural Biotechnology Institute (FABI), University of Pretoria, Pretoria, South Africa University of Pretoria Pretoria South Africa

**Keywords:** Anticancer activity, bioactive compounds, Dothideomycetes, Eurotiomycetes, extracellular enzymes, Sordariomycetes

## Abstract

*Usnea* is one of the largest and most diverse genera of fruticose lichens with global distribution. Endolichenic fungi, which thrive within lichen thalli, have emerged as a promising source of bioactive compounds, with the ability to synthesise a variety of metabolites with biopharmaceutical potential. In this study, four isolates of endolichenic fungi isolated from *Usnea* spp. were identified using comprehensive multi-gene phylogenetic analyses. These isolates were evaluated for their anticancer, antifungal, and antibacterial properties, as well as for their ability to produce extracellular enzymes. Our findings revealed that the isolates represent four novel species, named as *Amphisphaeriafalcata*, *Kirschsteiniotheliatumidula*, *Neoroussoellaannulata*, and *Veronaeabrunneicolor*. Our screening assay showed *N.annulata* and *V.brunneicolor* exhibited cytotoxic effects against the H460 human lung cancer cell line, with moderate inhibitory activity at a concentration of 100 μg/mL. The four fungal isolates exhibited distinct antifungal profiles against phytopathogens: *Amphisphaeriafalcata* specifically inhibited *Fusariumgraminearum*, while *Veronaeabrunneicolor* showed broad-spectrum activity against *Botrytiscinerea*, *F.graminearum*, and *Alternariaalternata*. No antibacterial effects were detected in any isolates. These fungi exhibited a diverse array of extracellular enzyme activities, including amylase, protease, gelatinase, glucose oxidase, and cellulase. Collectively, these results underscore the considerable biotechnological potential of endolichenic fungi as sources of bioactive compounds with applications in drug discovery, agriculture, and environmental management. These findings also highlight the ecological importance of endolichenic fungi, suggesting that they may play multifaceted roles in lichen symbioses and their environments. Continued exploration of these fungi is essential for unlocking their full pharmacological and industrial potential.

## ﻿Introduction

*Usnea* is one of the largest genera of fruticose lichens, currently ranking among the top ten most speciose genera with an estimated 350 described species with a global distribution (Lücking et al. 2020). This genus is infragenerically classified into the groups *Usneasensu stricto*, *Eumitria*, and *Dolichousnea*. *Usnea* can also be distinguished from the other two genera by the presence of usnic acid in the cortex and a central cartilaginous axis (Nadel and Clerc 2022). Moreover, various species of *Usnea* are widely valued for their medicinal properties. Thus, it has been a part of traditional medicine in various cultures. For example, over ten *Usnea* species in China are used as herbal remedies (Peng et al. 2012). The earliest recorded use of *Usnea* in traditional Chinese medicine dates back to 101 B.C., where it was referred to as “*Song Lo*” and used as an antimicrobial agent (Guo et al. 2008). *Usnea* exhibits a range of health benefits due to its production of various secondary metabolites, including antimicrobial, anticancer, antiproliferative, antioxidant, anti-inflammatory, antiulcer, hepatoprotective, and antigenotoxic properties (Paliya et al. 2016).

Secondary metabolites in lichens, including those from *Usnea*, are of fungal origin (Devashree et al. 2021; Sepahvand et al. 2021). Lichen-forming fungi are prolific producers of unique secondary metabolites, particularly phenolics, depsides, and depsidones (Devashree et al. 2021). Endolichenic fungi that proliferate within lichens are known to synthesise an assortment of metabolites with biopharmaceutical potential (Singh et al. 2017; Suryanarayanan and Thirunavukkarasu 2017; Agrawal et al. 2020). For instance, *Geotrichum* sp. and *Oidiodendron* sp. isolated from *Usnea* spp. have significant antimicrobial activity against pathogenic bacteria *Pantoeaagglomerans* and *Klebsiellapneumoniae*, respectively (Dumo et al. 2023). Santiago et al. (2021) showed that both lichens and endolichenic fungi possess antimicrobial properties. Lichen extracts were found to be effective against *Staphylococcusaureus* and *Candidaalbicans*. In contrast, endolichenic fungi extracts showed broader activity, targeting these organisms as well as *Escherichiacoli*. The wider spectrum of effectiveness exhibited by endolichenic fungi highlights their potential advantages in medicine and industry, particularly as they surpass the bioactivity of slower-growing lichens.

In addition to their diverse secondary metabolites, endolichenic fungi produce an array of extracellular enzymes, including amylases, cellulases, proteases, lipases, and laccases (Kannangara et al. 2009; Gan et al. 2023). Fungi isolated from lichens, such as *Parmotrema*, *Pseudocyphellaria*, and *Usnea* sp., including *Broomella* sp., *Curvularia* sp., *Nigrospora* sp., *Cladosporium* sp., *Chrysosporium* spp., *Phoma* sp., and *Penicillium* sp., exhibit varying enzymatic activities. These fungi, apart from *Cladosporium* sp. and *Curvularia* sp., possess amylase activity, while *Phoma* sp. secretes cellulase (Kannangara et al. 2009). Besides, several endolichenic fungal species, such as *Chaetomiumglobosum*, *Daldiniaeschscholtzii*, *Neofusicoccumoccultatum*, *Phanerochaetechrysosporium*, *Schizophyllumcommune*, and *Xylariafeejeensis*, show promise in biodegrading low-density polyethylene (Perera et al. 2022).

In our previous study, we successfully identified numerous endolichenic fungi from four species of *Usnea* (Si et al. 2023). Preliminary classification of these fungi using complete internal transcribed spacer (ITS) sequences indicated that some of these isolates displayed limited resemblance to known fungal species, hinting at potentially new taxa. Thus, to confirm this possibility, in this study, we amplified and sequenced additional gene regions and analysed them using phylogenetic approaches. We also explored the biotechnological and pharmaceutical potential of these fungi by evaluating the anticancer, antimicrobial, and enzymatic activity of these new fungal species.

## ﻿Materials and methods

### ﻿Fungal isolates

All fungal isolates used in this study were recovered in a previous study by Si et al. (2023). These isolates were retrieved from the microbial culture collection of Shandong Normal University and revitalised on potato dextrose agar medium (PDA; 46 g PDA powder [Qingdao Hope Bio-Technology Co., Ltd., China] and 1 L distilled water, pH 5.6 ± 0.2). The ex-type isolates of the new species described herein have been deposited at the China General Microbiological Culture Collection Centre (CGMCC) in Beijing, China. Corresponding type specimens, in the form of dry cultures, have been archived in the Herbarium Mycologicum at Academia Sinicae (HMAS), Beijing, China.

### ﻿DNA extraction, PCR amplification, and sequencing

The total genomic DNA was extracted from 14-day-old fungal cultures growing on PDA at 25 °C in darkness using a modified CTAB approach (Doyle and Doyle 1990). The complete ITS gene region was amplified and sequenced for each isolate. Subsequently, initial identification of the isolates was conducted using BLAST sequence similarity searches accessible through the NCBI GenBank. Following this preliminary identification, additional gene regions, including TEF, RPB2, LSU, SSU, and β-tubulin, specific to the taxonomic group of each fungal isolate, were amplified using the primers listed in Suppl. material 1: table S1.

PCR amplifications were conducted in 50 μL reactions, which included 1 μL DNA template, 2 μL each of forward and reverse primer (10 mM; Suppl. material 1: table S1), 19 μL of PCR-grade water, and 25 μL of 1-5TM2 High-Fidelity Master Mix (Tsingke Biotech Co., China). The PCR protocol for all gene regions was initial denaturation at 94 °C for 3 min, 30 cycles of 94 °C for 30 s, 55 °C for 1 min, 72 °C for 1 min, and final extension at 72 °C for 10 min. Agarose gel electrophoreses were used to confirm positive amplifications. Sangon Biotech Company (Shanghai, China) purified and sequenced all PCR products. The resulting forward and reverse sequences were assembled using Geneious v.10.2.2 (Biomatters, Auckland, New Zealand). All the sequences were submitted to the NCBI GenBank (Suppl. material 1: tables S2–S5).

### ﻿Sequence alignment and phylogenetic analyses

During preliminary identification using sequence similarity searches using complete ITS gene region, four isolates, CGMCC3.23740, CGMCC3.23629, CGMCC3.23625, and CGMCC3.23628, were identified as potential novel taxa, closely related to the genera *Amphisphaeria* (Amphisphaeriaceae, Sordariomycetes), *Kirschsteiniothelia* (Kirschsteiniotheliaceae, Dothideomycetes), *Roussoella*/*Neoroussoella* (Thyridariaceae, Dothideomycetes), and *Veronaea* (Herpotrichiellaceae, Eurotiomycetes), respectively. Thus, individual gene datasets were compiled for each genus based on previous studies: *Amphisphaeria* (Suppl. material 1: table S2, ITS, LSU, and RPB2) (Li W-L et al. 2024), *Kirschsteiniothelia* (Suppl. material 1: table S3, ITS, LSU, and SSU) (Xu et al. 2023), and *Veronaea* (Suppl. material 1: table S4, ITS, LSU, SSU, and β-tubulin) (Su et al. 2023). To identify the isolate closely as *Roussoella*/*Neoroussoella*, we retrieved ITS, LSU, SSU, TEF, and RPB2 sequences of all validly described species from Thyridariaceae listed in the NCBI GenBank Taxonomy (https://www.ncbi.nlm.nih.gov/taxonomy; Suppl. material 1: table S5). All these datasets included sequences from the novel taxa identified in this study and sequences from ex-type isolates. If sequences from an ex-type isolate for a species were unavailable, then sequences from an alternative isolate were considered.

All datasets were aligned using MAFFT v. 7.407 (Katoh et al. 2019) and manually adjusted using MEGA v. 10.2.0 (Kumar et al. 2018). During the preliminary phylogenetic analyses of the single gene datasets, ITS sequences from two fungal species from Thyridariaceae, *Parathyridariaflabelliae* (MUT 4859; KR014355) and *Roussoellopsismacrospora* (MFLUCC 12-0005; KJ739604), emerged as misidentified taxa. Thus, it was removed from the final dataset for all gene regions.

Final concatenated datasets were analysed using the maximum likelihood (ML), Bayesian inference (BI), and maximum parsimony (MP) approaches. jModelTest v. 2.1.6 (Darriba et al. 2012) was used to identify suitable nucleotide substitution models. ML analyses were performed using RAxML v. 8.2.12 (Stamatakis et al. 2008) with the substitution model GTR+GAMMA and 1000 bootstrap replications. For BI analysis, MrBayes v. 3.2.7 (Ronquist and Huelsenbeck 2003) with four MCMC chains was run for 5 M generations from a randomly chosen starting tree with the stop value set at 0.01, the temperature set at 0.2, and trees sampled every 100 generations. A quarter of the trees were discarded as burn-in, and the remaining were used to build majority rule consensus trees. MP analyses were done using MEGA with 1,000 bootstrap replications, and gaps were considered the fifth state character. The resulting phylogenetic trees were visualised using FigTree v. 1.4.4 (http://tree.bio.ed.ac.uk/software/figtree/). All the alignments and trees were submitted to the Mendeley Data (https://doi.org/10.17632/fbwj88c2nb.1).

### ﻿Morphological characterisation

All isolates were sub-cultured on malt extract agar (MEA, 20 g agar, 20 g malt extract [Qingdao Hope Bio-Technology Co., Ltd., Shandong, China], 1 L deionised water), oatmeal agar (OA; 30 g oatmeal, 15 g agar, 1 L distilled water, pH 7.2 ± 0.2), synthetic nutrient-poor agar (SNA, 1g KH_2_PO_4_, 1g KNO_3_, 0.5 g MgSO_4_ ·7H_2_O, 0.5 g KCl, 0.2 g glucose, 0.2 g sucrose, 20 g agar, 1 L deionised water) and PDA. All Petri plates were incubated at 25 °C for 40 d. If no sporulating structures were observed after incubation, autoclaved pine needles and dried lichen pieces were added to all the above-mentioned media. Micro-morphological characters such as hyphae, conidia, and conidiophores were photographed and measured (n = 50) using a Leica DFC495 camera attached to a Leica DM6 microscope. ImageJ v. 1.54h (Collins 2007) was used for measuring the taxonomically relevant structures.

### ﻿Growth studies

Agar plugs (5 mm in diameter) covered with mycelium were excised from the actively growing edges of one-week-old cultures of each isolate and placed at the centre of 90 mm Petri plates containing 2% MEA. Three replicate plates were used for each temperature within the range of 5–35 °C at an interval of 5 °C to determine the optimal growth temperatures. The Petri plates were incubated in the dark. Colony diameters were measured every two days until the hyphae reached the edges of the Petri dishes or up to the eighth day.

### ﻿Biological activity assay

#### ﻿Preparation of fungal extracts

All fungal isolates were sub-cultured on PDA medium and incubated at 25 °C until the hyphal growth extended to the edges of the Petri dishes. Upon reaching this stage of growth, both the mycelia and PDA medium were harvested and macerated using separate sterile mortars and pestles. Subsequently, the mycelial mass and PDA medium were subjected to sequential extractions using 400 mL of ethyl acetate (EA, Tianjin Fuyu Fine Chemical Co., Ltd., China). The organic phase obtained was concentrated under a vacuum to eliminate the solvent, resulting in the crude extract. The dried crude extracts were then reconstituted in 100% dimethyl sulfoxide (DMSO; Solarbio, China) to prepare them for use in subsequent analyses.

#### ﻿Anticancer assay

H460 and A549 are widely used non-small cell lung cancer (NSCLC) cell lines for anticancer research, offering distinct characteristics for comparative analysis with differences in sensitivity, reflecting their unique molecular traits (Gomathinayagam et al. 2008; Tsui et al. 2014; Heavey et al. 2018). Therefore, these two cell lines were chosen for the anticancer assay. To measure the cytotoxicity against H460 cells, a 3-(4,5-dimethylthiazol-2-yl)-2,5-diphenyl-2H-tetrazolium bromide (MTT) colorimetric assay was used following the protocol suggested by Xie et al. (2016). Cells (6,000/well) were seeded into 96-well plates and incubated at 37 °C with 5% CO_2_. Following this, the cells were treated with either a control (vehicle, 100% DMSO) or 100 µg/mL of crude extracts and incubated for 24 h, and then the cells were incubated with MTT (Sigma-Aldrich, the USA) for 4 h in the dark. A plate reader (Bio-Rad Laboratories, Richmond, CA) was used to measure light absorbance at 570 nm, determining the cell growth response to the crude extract. Extracts that resulted in a cell death rate greater than 60% were selected for further testing on both H460 and A549 cell lines, using 50 µg/mL of crude extracts.

Cytotoxicity data were analysed using one-way ANOVA with Tukey’s post-hoc test for comparisons at 100 μg/mL and unpaired t-tests for the 50 μg/mL assays available through R. Differences were considered significant at p < 0.05. IC_50_ values were determined using the IC_50_ Calculator available through https://www.aatbio.com/tools/ic50-calculator.

#### ﻿Antimicrobial assay

All fungal isolates were inoculated into Erlenmeyer flasks containing 50 mL of potato dextrose broth (PDB) and incubated at 25 °C for seven days in the dark. The cell-free culture filtrate was sterilised by filtering through a 0.22 μm pore size filter (BKMAM, China). Antifungal assays were conducted according to the method described by Li X et al. (2022). One mL of sterile filtrate was added to 9 mL of PDA medium poured into Petri plates, resulting in a final concentration of 10%. Undiluted PDA plates were used for the blank control. Mycelial agar plugs (6 mm in diameter) of *Botrytiscinerea* (isolated from tomato), *Fusariumgraminearum* (isolated from wheat), and *Alternariaalternata* (isolated from grape) were placed at the centre of the Petri plates and incubated for seven days. The PDA agar plate without any sterile filtrate served as the control. All plates were cultured at 25 °C, and the colony diameter of the fungal pathogens was measured after seven days. The inhibition rate was calculated using the formula inhibition rate (%) = 100 × (C−T)/C, where C = the radial growth of the control (mm) and T = the radial growth of the test culture (mm).

The disc diffusion method was used to assess antibacterial activity. *Escherichiacoli* (ATCC 51446) and *Staphylococcusaureus* (ATCC 29213) were streaked onto LB (lysogeny broth, Solarbio, China) agar plates and incubated at 37 °C for 12 hours. A single colony was transferred into 50 mL LB broth in Erlenmeyer flasks and incubated overnight at 37 °C with shaking at 180 rpm. The resulting cell suspensions were used immediately to evaluate antibacterial activity. One mL of the diluted microbial culture (1–2 × 10^5^ colony-forming units (cfu)/mL) was uniformly distributed into 9 mL of LB-agar medium and poured into Petri dishes. Ten μL of cell-free culture filtrate were dripped onto 6mm filter discs and placed on the surface of the LB-agar medium. For the negative controls, 10 μL of sterile water was used, while the positive control consisted of penicillin (100 mg/mL; Sangon, China). The agar plates were incubated at 37 °C for 24 hours, and the inhibition zones around the discs were measured.

### ﻿Extracellular enzyme assay

The screening medium for amylase consisted of 20 g agar (Solarbio, China), 2.5 g soluble starch (Damao, China), 2.5 g (NH_4_)_2_SO_4_, 3 g KH_2_PO_4_, 0.25 g CaCl_2_·6H_2_O, 5 g peptone (Solarbio, China), and 1 L deionised water. Fungal isolates were inoculated onto the medium and incubated for 4–7 days at 25 °C. After adding iodine, a transparent zone around the fungal colony indicated amylase activity.

The cellulase screening medium included 0.5 g KH_2_PO_4_, 0.3 g MgSO_4_·7H_2_O, 1.88 g carboxymethyl cellulose-Na, 0.2 g Congo red, 20 g agar (Solarbio, China), and 1 L deionised water. Fungi were inoculated and incubated for 4–7 days at 25 °C. The presence of a transparent zone around the fungal colony indicated cellulose activity.

For glucose oxidase screening, the medium contained 80 g glucose, 3 g peptone (Solarbio, China), 2 g KH_2_PO_4_, 0.7 g MgSO_4_·7H_2_O, 0.5 g KCl, 4 g NaNO_3_, 3.5 g CaCO_3_, 10 g soluble starch, 0.3 g sodium deoxycholate, 1.7 g KI, 20 g agar (Solarbio, China), and 1 L deionised water. Fungi were inoculated and incubated for 4–7 days at 25 °C. A purple zone around the fungal colony indicated glucose oxidase activity.

The protease screening medium included 15 g skim milk, 3 g beef extract, 5 g NaCl, 10 g peptone (Solarbio, China), 20 g agar (Solarbio, China), and 1 L deionised water, with a pH of 7.2–7.5. The medium was autoclaved for 20 minutes at 121 °C. Fungi were inoculated and incubated for 4–7 days at 25 °C. A transparent zone around the fungal colony indicated protease activity.

The gelatinase screening medium consisted of 5 g NaCl, 10 g peptone (Solarbio, China), 3 g beef extract, and 120 g gelatin. This medium was autoclaved at 110 °C for 20 minutes. Fungi were inoculated and incubated for 4–7 days at 25 °C, followed by refrigeration at 4 °C overnight. Liquefaction after overnight refrigeration indicated gelatinase activity.

## ﻿Results

### ﻿Phylogenetic analyses

**Figure 1.**
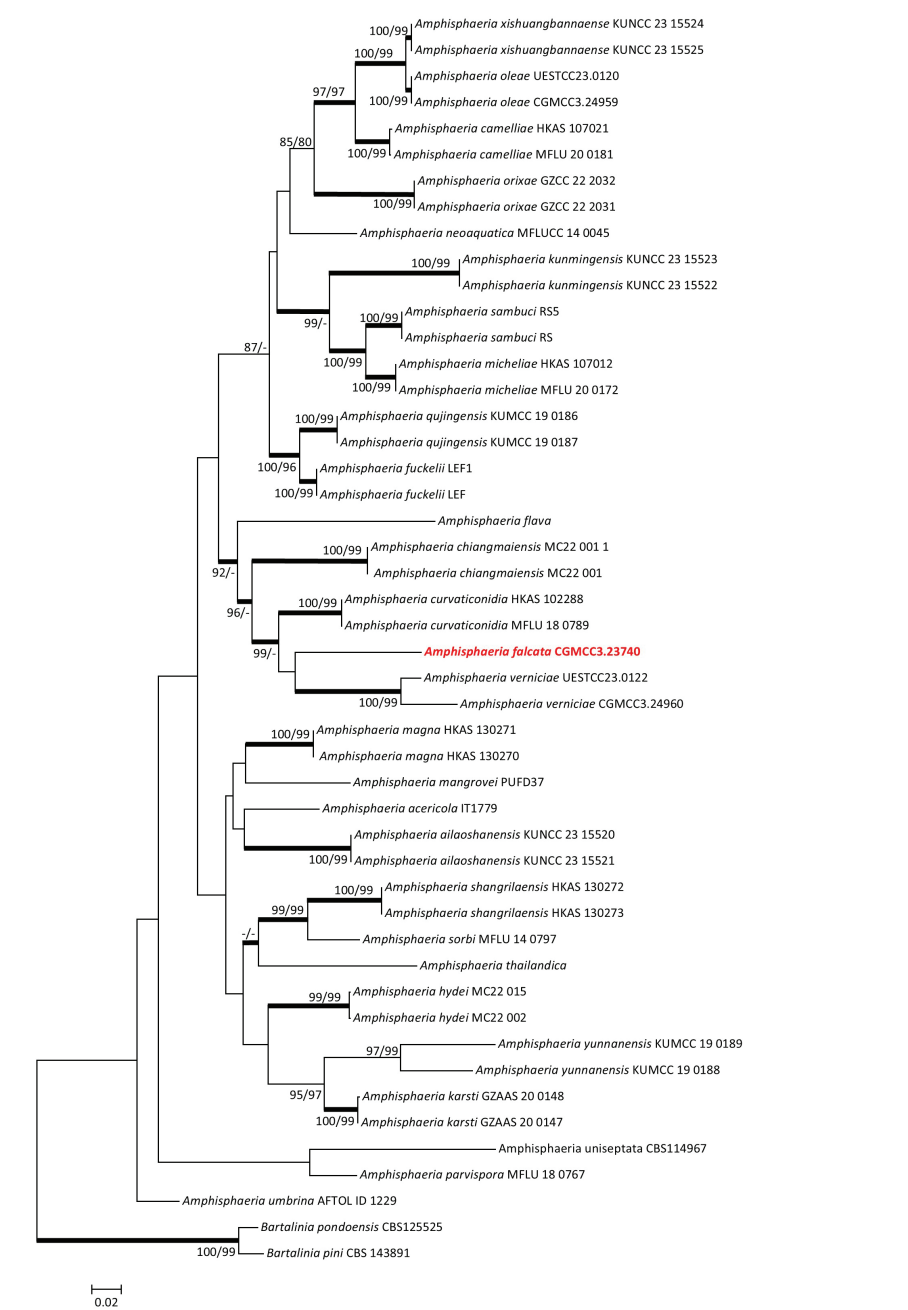
Maximum likelihood tree of *Amphisphaeria* species constructed using the concatenated dataset (LSU+ITS+RPB2+β-tubulin). Bootstrap support values ≥ 75% are indicated above the nodes as ML/MP, and posterior probabilities ≥ 0.90 are indicated by bold branches. Isolates obtained in this study are in bold font.

Four gene regions, including LSU, ITS, RPB2 and β-tubulin, were used to identify CGMCC3.23740. The concatenated dataset included 38 sequences with 3176 base pairs (LSU: 1–893, ITS: 894–1586, RPB2: 1587–2279, β-tubulin: 2280–3072). CGMCC3.23740 emerged as a sister to *Amphisphaeriaverniciae* but without strong support in concatenated trees (Fig. 1). Therefore, CGMCC3.23740 was identified as a new species in *Amphisphaeria*.

**Figure 2.**
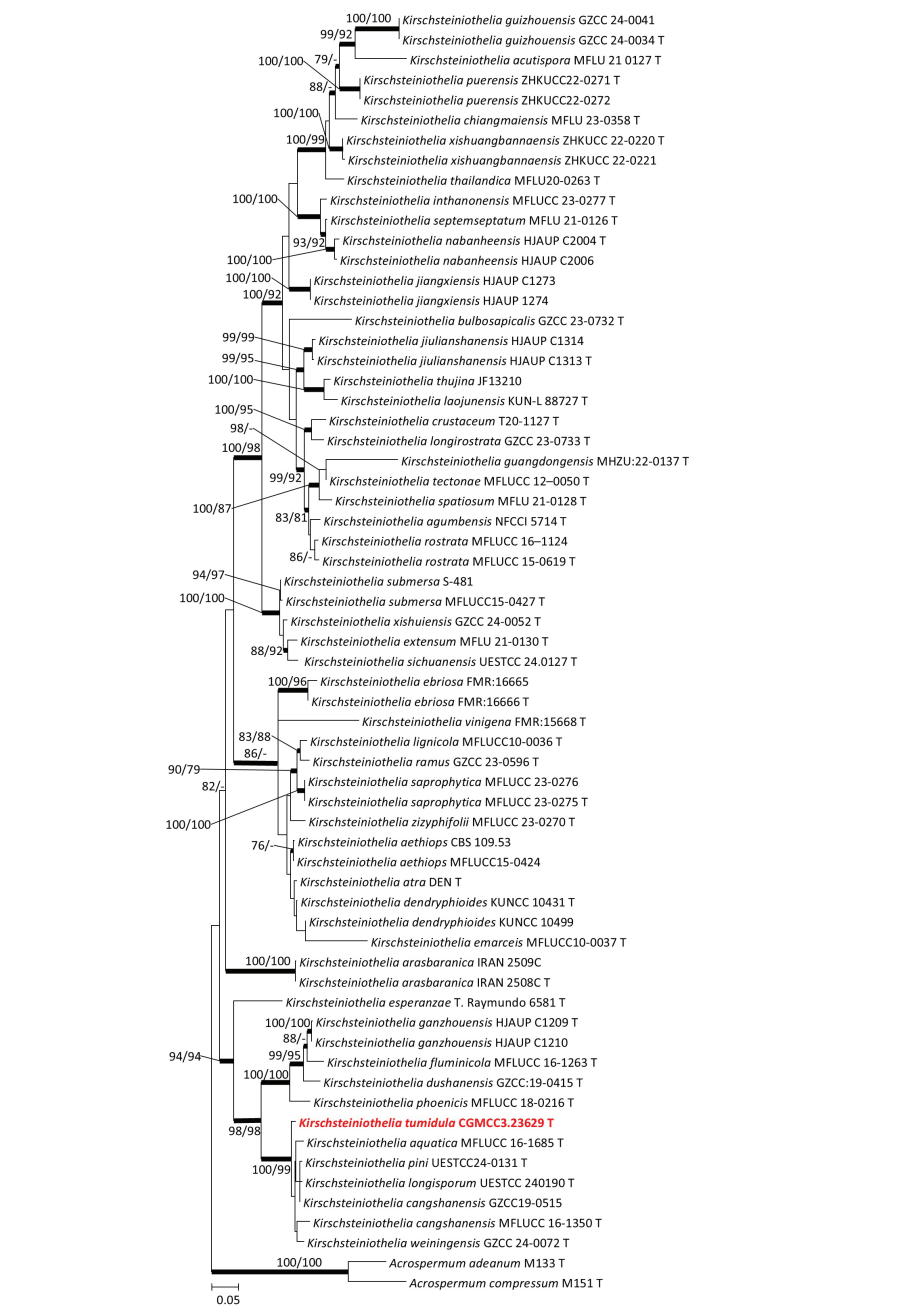
Maximum likelihood tree of *Kirschsteiniothelia* species constructed using the concatenated dataset (SSU+ITS+LSU). Bootstrap support values ≥ 75% are indicated above the nodes as ML/MP, and posterior probabilities ≥ 0.90 are indicated by bold branches. Isolates obtained in this study are in bold font.

The concatenated dataset used to identify CGMCC3.23629 includes 64 taxa and 2347 characters, including gaps (SSU: 1–900; ITS: 901–1507; LSU: 1508–2347). In the concatenated tree, CGMCC3.23629 is nested within a clade that included *Kirschsteiniotheliaaquatica*, *K.cangshanensis*, *K.longisporum*, *K.pini* and *K.weiningensis* with support of ML/MP/BI: 100/99/1 (Fig. 2). Therefore, CGMCC3.23629 was identified as a new species in the genus *Kirschsteiniothelia*.

**Figure 3.**
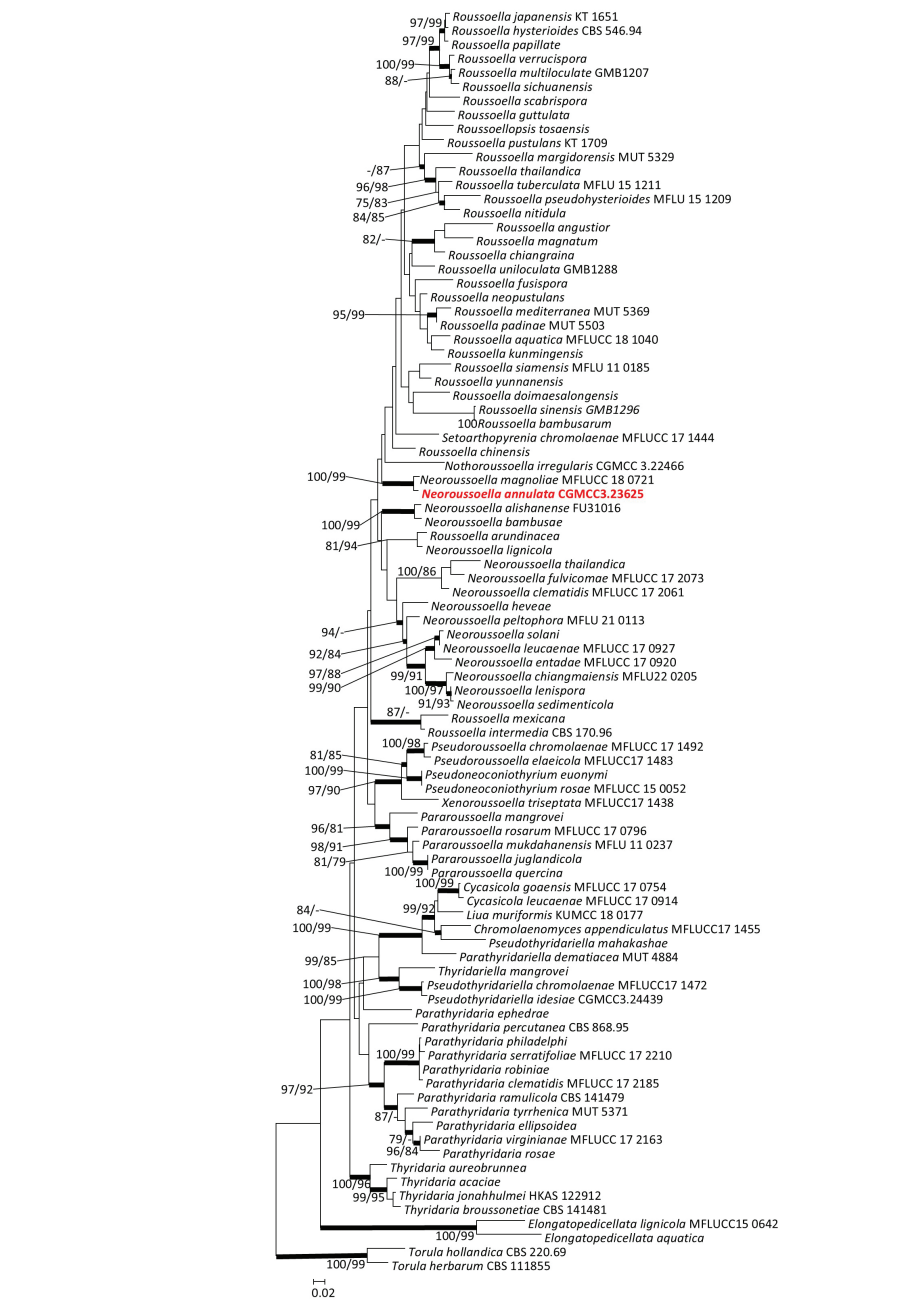
Maximum likelihood tree of Thyridariaceae species constructed using the concatenated dataset (SSU+ITS+LSU+TEF+RPB2). Bootstrap support values ≥ 75% are indicated above the nodes as ML/MP, and posterior probabilities ≥ 0.90 are indicated by bold branches. Isolates obtained in this study are in bold font.

The concatenated dataset used to identify CGMCC3.23625 includes 90 taxa and 3909 characters, including gaps (SSU: 1–1005; ITS: 1006–1429; LSU: 1430–2273; TEF: 2274–2999; RPB2: 3000–3909). CGMCC3.23625 was grouped with *Neoroussoellamagnoliae* outside of *Roussoella* without strong support in the concatenated ML trees (Fig. 3). Therefore, CGMCC3.23625 was identified as a novel *Neoroussoella* species in the family Thyridariaceae.

**Figure 4.**
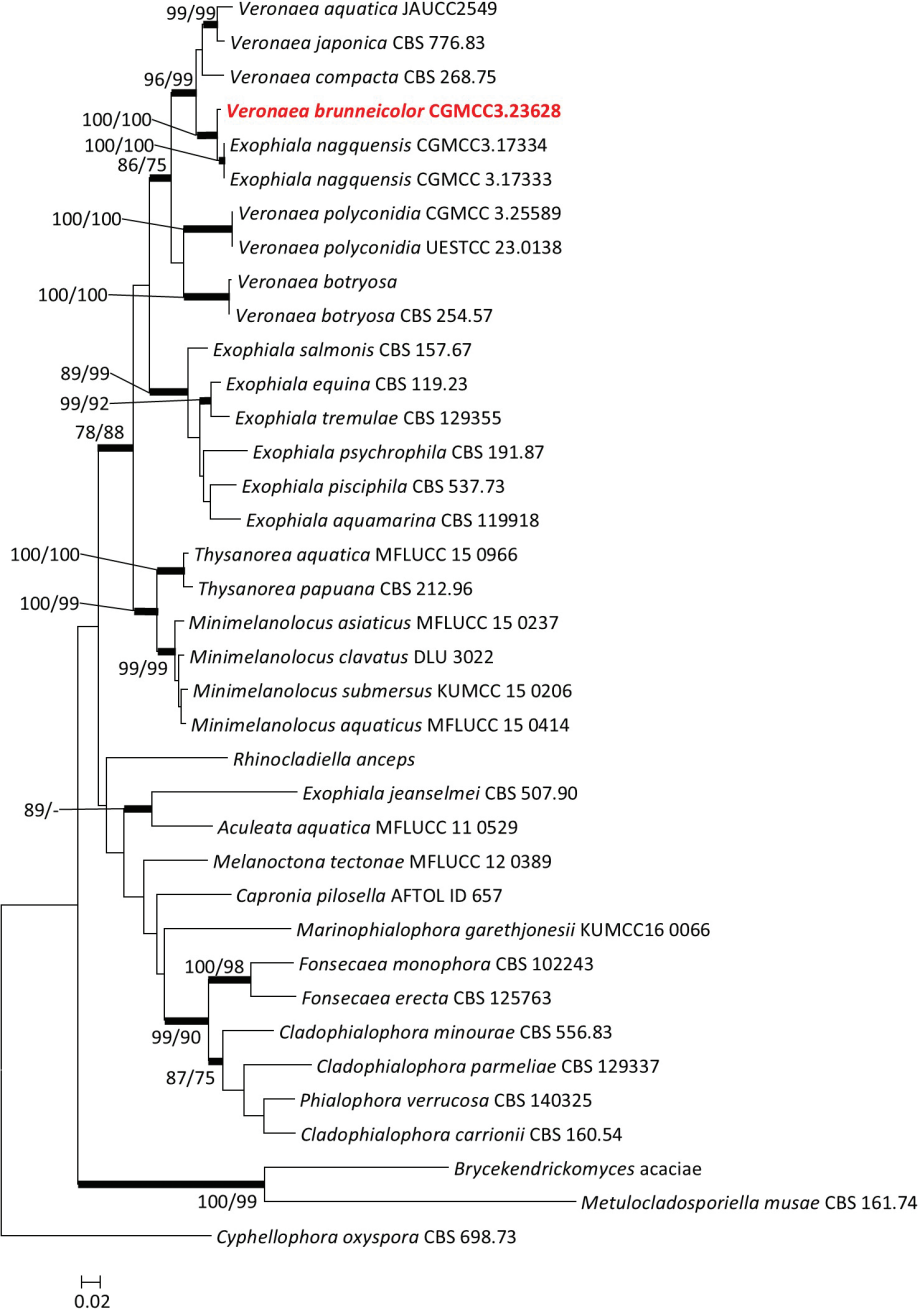
Maximum likelihood tree of *Veronaea* and related species constructed using the concatenated dataset (SSU+ITS+LSU+β-tubulin). Bootstrap support values ≥ 75% are indicated above the nodes as ML/MP, and posterior probabilities ≥ 0.90 are indicated by bold branches. Isolates obtained in this study are in bold font.

The concatenated dataset used to identify CGMCC3.23628 includes 37 taxa and 2877 characters, including gaps (SSU: 1–977; ITS: 978–1676; LSU: 1677–2513; β-tubulin: 2514–2877). In the concatenated tree, CGMCC3.23628 formed a sister clade with *Exophialanagquensis* inside the genus *Veronaea* with strong support (ML/MP/BI: 100/100/1) (Fig. 4). Therefore, CGMCC3.23628 was identified as a new species in the genus *Veronaea*.

### ﻿Taxonomy

#### ﻿ Amphisphaeria falcata

Taxon classificationFungiAmphisphaerialesAmphisphaeriaceae

H.L. Si, R.L. Chang, T. Bose & Y. C. Wang sp. nov.

13CC7BBB-E518-5F1D-87A3-DC93188F1A22

847894

Fig. 5a–i

##### Etymology.

The name refers to the sickle-shaped conidia.

##### Type.

China • Yunnan Province, Chuxiong Yi Autonomous Prefecture, Chuxiong City, Dayao County (26°32'71.54"N, 100°57'3.6"E), isolated from the medullary tissue of the lichen *Usneadiffracta* (SDCX40), 13 Nov. 2020, H. L. Si, CX40A8 = CGMCC3.23740 (the ex-holotype culture), dried culture HMAS 352144 (holotype specimen), GenBank Accession Numbers: ITS OQ645270; LSU OQ645284; SSU OQ625477; RPB2 OQ696281; and β-tubulin OQ696283.

##### Description.

**Figure 5.**
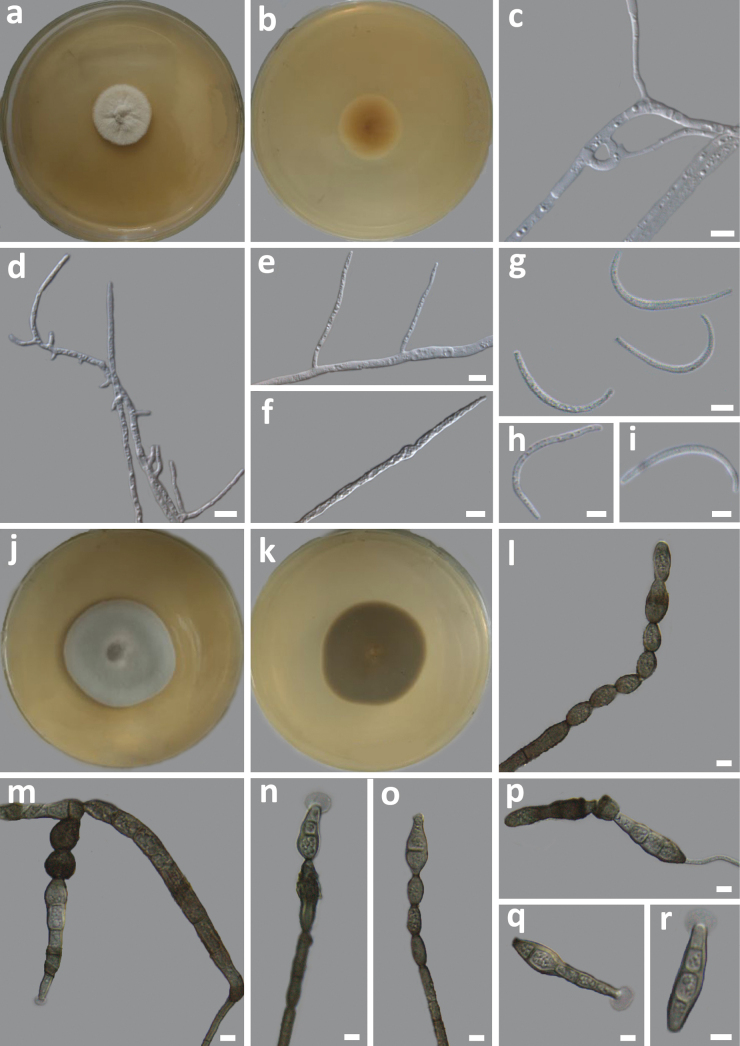
Morphology of *Amphisphaeriafalcata* sp. nov. (HMAS 352144) **a** top and **b** reverse view of a 10-day-old culture growing on a PDA**c–f** hyphae **g–i** conidia; *Kirschsteiniotheliatumidula* sp. nov. (HMAS 352146) **j** top and **k** reverse view of a 15-day-old culture growing on a PDA**l** conidiophore **m–o** conidiophore with conidia **p** germinating conidia **q, r** conidia. Scale bars: 5 μm.

***Hyphae*** smooth, grey, septate, septa inconspicuous, compartments cylindrical, branched, measuring 1.19−3.15 μm (x̄ = 1.93 μm, n = 50) in diam (Fig. 5c–f). ***Conidia*** hyaline, surface smooth, sickle-shaped, 25.07−42.22 × 1.12−2.43 μm (x̄ = 31.83 × 1.76 μm, n = 11) in diameter (Fig. 5g–i). No sexual morph was observed.

##### Culture characteristics.

On PDA, after 10 days of incubation at 25 °C, the colonies were white in colour, velvety on the surface, slightly raised in the centre, with an entire margin and irregular radial folds (Fig. 5a), and the reverse was yellow in the centre with a white halo around the margin (Fig. 5b). The optimum growth temperature was 25 °C (1.42 mm/day). No growth was detected at 5 °C and 35 °C.

##### Host.

*Usneadiffracta*.

##### Distribution.

Yunnan, China.

##### Note.

*Amphisphaeriafalcata* is phylogenetically close to *A.verniciae*. However, the sexual state of *A.falcata* has not been observed, and the conidia of *A.verniciae* have not been observed. There is a significant variation, a total of 297 bps, in the ITS, LSU, β-tubulin, and RPB2 gene sequences between these two species (ITS 71 bps, LSU 15 bps, β-tubulin 162 bps, RPB2 49 bps).

#### ﻿ Kirschsteiniothelia tumidula

Taxon classificationFungiPleosporalesKirschsteiniotheliaceae

H.L. Si, R.L. Chang, T. Bose & Y. C. Wang sp. nov.

ED033CC6-D0B4-5759-B8C8-D8B422B9372E

847896

Fig. 5j–r

##### Etymology.

The name refers to the convex colony of this fungus on PDA.

##### Type.

China • Yunnan Province, Chuxiong Yi Autonomous Prefecture, Chuxiong City, Dayao County (26°32'71.54"N, 100°57'3.6"E), isolated from the medullary tissue of the lichen *Usneaaciculifera* (SDCX79), 13 Nov. 2020, H. L. Si, CX79B2 = CGMCC3.23629 (the ex-holotype culture), dried culture HMAS 352146 (holotype specimen), GenBank Accession Numbers: ITS OQ645272; LSU OQ645286; SSU OQ645279.

##### Description.

***Hyphae*** smooth, olivaceous in colour, branched, septate, measuring approximately 1.78–4.76 μm (x̄ = 2.61, n = 50) in diam (Fig. 5i−o). ***Conidiophores*** macronematous, mononematous, terminal or lateral, pale blue to olive green, smooth, unbranched, septate, measuring 34.07–70.85 × 5.11–10.06 μm (x̄ = 50.63 × 7.17 μm, n = 11) (Fig. 5m–o). ***Conidiogenous cells*** monoblastic, integrated, terminal, determinate, spherical or ovoid in shape, surface smooth, pale blue to olive green in colour, measuring 4.95–11.61 × 4.39–10.51 μm (x̄ = 8.82 × 6.14 μm, n = 11) (Fig. 5m−p). ***Conidia*** acrogenous, solitary, dry, pale olivaceous, pale at apex, septate, with 1–4 septa, slightly constricted at septum, obclavate, rostrate, smooth, straight or slightly curved, truncate at base, sometimes with a gelatinous sheath surrounding the apex, measuring 7.23–38.00 × 3.92–11.30 μm (x̄ = 6.64 × 18.29 μm, n = 50) (Fig. 5m–o). No sexual morph was observed.

##### Culture characteristics.

On PDA, after 7 days of incubation at 25 °C, the colony is pale bluish green in the centre, the edge is grey, the centre is raised, the surface is tomentose, and the margin is entire (Fig. 5j). The reverse of the colony is brown (Fig. 5k). The optimal growth temperature is 25 °C (5.86 mm/day). Slow growth (1.42 mm/day) was observed at 5 °C, and no growth was detected at 30 °C and 35 °C.

##### Host.

*Usneaaciculifera*.

##### Distribution.

Yunnan, China.

##### Note.

*Kirschsteiniotheliatumidula* is phylogenetically close to *K.aquatica*, *K.cangshanensis*, *K.longisporum*, *K.pini*, and *K.weiningensis*. However, the *Conidiophores* of *K.tumidula* ranges between 34.07–70.85 μm are shorter than those of *K.aquatica*, *K.cangshanensis*, *K.longisporum*, *K.pini*, and *K.weiningensis* which range between 105.5–135.5 μm, 114–151 μm, 115–285 μm, 69–124 μm, and 75–125 μm, respectively (Bao et al. 2018; Jin et al. 2024; Tian et al. 2024; Xiao et al. 2025). Both *K.tumidula*, *K.cangshanensis*, and *K.weiningensis* exhibit a gelatinous sheath surrounding their apices, whereas *K.aquatica*, *K.longisporum*, and *K.pini* lack this feature. In addition, there is a significant variation in the ITS and LSU among these three species. There was a total of 37 bps (ITS: 34 bps, LSU: 3 bps, SSU: 0 bps) differences between *K.tumidula* and *K.aquatica*, 45 bps (ITS: 43 bps, LSU: 2 bps, SSU: 0 bps) differences between *K.tumidula* and *K.cangshanensis*, and 35 bps (ITS: 29 bps, LSU: 5 bps, SSU: 1 bps) differences between *K.tumidula* and *K.longisporum*, 39 bps (ITS: 36 bps, LSU: 3 bps, SSU: 0 bps) differences between *K.tumidula* and *K.pini*, and 43 bps (ITS: 36 bps, LSU: 6 bps, SSU: 1 bps) differences between *K.tumidula* and *K.weiningensis*.

#### ﻿ Neoroussoella annulata

Taxon classificationFungiPleosporalesRoussoellaceae

H.L. Si, R.L. Chang, T. Bose & Y. C. Wang sp. nov.

48A4820F-B0FA-56F8-A2B1-03B8B5C39274

847891

Fig. 6a–h

##### Etymology.

The name refers to the zonate colony morphology on PDA.

##### Type.

China • Yunnan Province, Chuxiong Yi Autonomous Prefecture, Chuxiong City, Dayao County (26°32'71.54"N, 100°57'3.6"E), isolated from the medullary tissue of the lichen *Usneaceratina* (SDCX26), 13 Nov. 2020, H. L. Si, CX26A1B = CGMCC3.23625 (the ex-holotype culture), dried culture HMAS 352142 (holotype specimen), GenBank Accession Numbers: ITS OQ645267; LSU OQ645281; SSU OQ645274; RPB2 OQ696278; TEF1 OQ696275.

##### Description.

**Figure 6.**
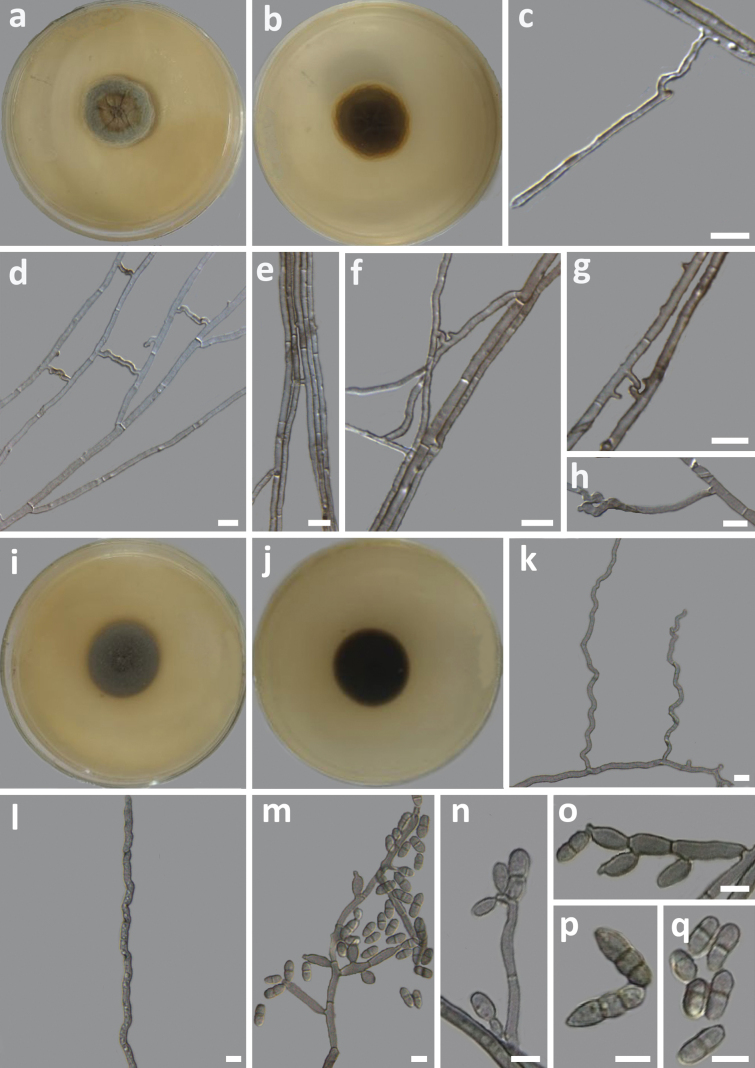
Morphology of *Neoroussoellaannulata* sp. nov. (HMAS 352142) **a** top and **b** reverse view of a 15-day-old culture growing on a PDA**c–h** hyphae; *Veronaeabrunneicolor* sp. nov. (HMAS 352145) **i** top and **j** reverse view of a 15-day-old culture growing on a PDA**k, l** hyphae; **m–o** conidiophore with conidia **p, q** conidia. Scale bars: 5 μm.

***Hyphae*** smooth, pale coloured to brown, branched, septate, measuring 0.94−2.99 μm (x̄ = 1.48 μm, n = 50) in diam (Fig. 6c–h). Often, two parallel hyphae form hyphal anastomosis (Fig. 6d, g). No spores or sexual morphs were observed.

##### Culture characteristics.

On PDA, after 7 days of incubation at 25 °C, the colony has a light brown centre with curved elevations, surrounded by a greyish green concentric circle, and the margin is light grey. The surface is rough with short, fine hairs, and the edges are smooth and irregularly cracked (Fig. 6a). The reverse of the colony has a dark brown centre and edges that range from brown to yellow (Fig. 6b). The optimal growth temperature is 25 °C (1.57 mm/day). No growth was detected at 5 °C and 35 °C.

##### Host.

*Usneaceratina*.

##### Distribution.

Yunnan, China.

##### Note.

*Neoroussoellaannulata* is closely related to *N.magnoliae* in both multigene and single-gene phylogenetic analyses. We didn’t observe any reproductive structures for *N.annulata*; however, the sexual morph of *N.magnoliae* is known (Yuan et al. 2020). A significant genetic divergence was observed between the species, with a total of 50 base pair differences in the sequences analysed: ITS (19 bps), SSU (21 bps), LSU (0 bps), and TEF (10 bps). Furthermore, the ecological preferences of these species are distinct; *N.annulata* was isolated from *U.ceratina*, whereas *N.magnoliae* is a saprobic species isolated from dead twigs of *Magnolia* species (Yuan et al. 2020).

#### ﻿ Veronaea brunneicolor

Taxon classificationFungiChaetothyrialesHerpotrichiellaceae

H.L. Si, R.L. Chang, T. Bose & Y. C. Wang sp. nov.

9F7C7388-0796-55E0-880F-7E8D1D307720

847895

Fig. 6i–q

##### Etymology.

The name refers to the brown colony morphology on PDA.

##### Type.

China • Yunnan Province, Chuxiong Yi Autonomous Prefecture, Chuxiong City, Dayao County (26°32'71.54"N, 100°57'3.6"E), isolated from the medullary tissue of the lichen *Usneaaciculifera* (SDCX68), 13 Nov. 2020, H. L. Si, CX68C101 = CGMCC3.23628 (the ex-holotype culture), dried culture HMAS 352145 (holotype specimen), GenBank Accession Numbers: ITS OQ645271; LSU OQ645285; SSU OQ645278; β-tubulin OQ696284.

##### Description.

***Hyphae*** smooth, bluish-gray, septate, branched, measuring 1.24–3.75 μm (x̄ = 2.57 μm, n = 50) in diam (Fig. 6k, l). ***Conidiophores*** arising laterally on hyphae, bluish-gray, septate, branched or unbranched, sometimes reduced to *conidiogenous cells*, measuring 11.43–83.85 μm (x̄ = 38.50 μm, n = 10) (Fig. 6m–o). ***Conidiogenous cells*** holoblastic, polyblastic, integrated, terminal, determinate, cylindrical or ovoid, geniculate, smooth, grey, measuring 8.42–40.56 × 1.78–4.29 μm (x̄ = 15.46 × 2.77 μm, n = 50) (Fig. 6m–o). ***Conidia*** solitary and smooth, cylindrical to ellipsoid, straight or slightly curved, apex obtuse, base acute with a prominent scar, pale brown in colour, usually uniseptate, rarely bi-septate, often constricted at septa, measuring 4.12–12.09 × 1.96–3.05 μ (x̄ = 6.72 × 2.43 μm, n = 50) (Fig. 6m–q). No sexual morph was observed.

##### Culture characteristics.

On PDA, after 7 days of incubation at 25 °C, the colony gray-brown in colour, flat, with a tomentose surface, margin entire, and partially immersed in the medium (Fig. 6i). The reverse of the colony is dark brown (Fig. 6j). The optimal growth temperature is 25 °C (1.26 mm/day). No growth was detected at 5 °C and 35 °C.

##### Host.

*Usneaaciculifera*.

##### Distribution.

Yunnan, China.

##### Note.

Our phylogenetic analysis showed that all *Veronaea* species, including our isolates and the type species *V.botryose*, form a well-supported monophyletic clade distinct from *Exophiala*, supporting the placement of *V.brunneicolor* within *Veronaea*. Morphologically, our isolate also differs from *Exophiala* by producing solitary conidia (vs. slime-aggregated) and having holoblastic-polyblastic conidiogenous cells (vs. ampulliform) (Carmichael 1967). Although *E.nagquensis* clustered within *Veronaea*, its separation from *E.salmonis*, the type species of *Exophiala*, suggests plausible misidentification. *Veronaeabrunneicolor* is phylogenetically close to *E.nagquensis*. However, the conidia diam *of V.brunneicolor* is shorter than *E.nagquensis* (2.43 μm vs. 3.3 μm) (Sun et al. 2020). Conidia of *V.brunneicolor* are uni- or bi-septate, whereas in *E.nagquensis*, they are aseptate. In addition, there was a total of 15 bps differences between *V.brunneicolor* and *E.nagquensis* in ITS (6 bps), LSU (2 bps), SSU (0 bps) and β-tubulin (7 bps) sequences.

### ﻿Biological activity assay

#### ﻿Anticancer assay

**Figure 7.**
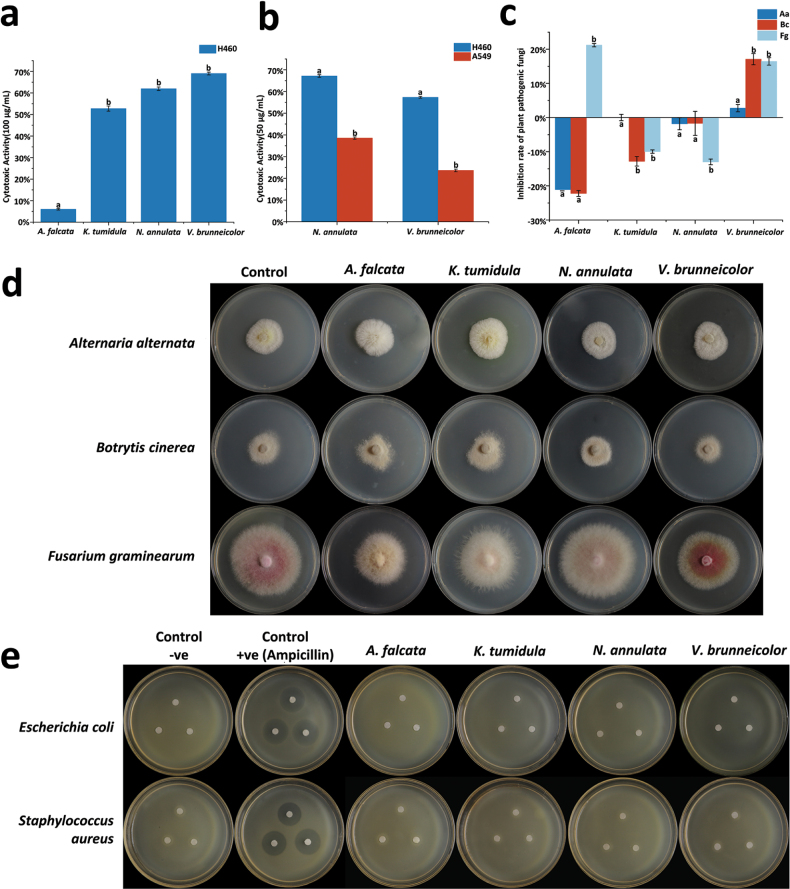
Anticancer, antifungal, and antibacterial activity of *Amphisphaeriafalcata* (CGMCC3.23740), *Kirschsteiniotheliatumidula* (CGMCC3.23629), *Neoroussoellaannulata* (CGMCC3.23625), and *Veronaeabrunneicolor* (CGMCC3.23628) **a, b** the anticancer activity **c, d** the antifungal activity **e** the antibacterial activity. All experiments were performed in triplicate and repeated once.

In evaluating the cytotoxic activity of crude extracts from endolichenic fungi at 100 μg/mL against the H460 cell line, *V.brunneicolor*, *N.annulata*, and *K.tumidula* exhibited significantly higher cytotoxicity, inhibiting cell growth by 69.05%, 62.01%, and 52.75%, respectively. These values were significantly greater (p < 0.05) than those of *A.falcata* (6.03%) (Fig. 7a). Based on this activity, *V.brunneicolor* and *N.annulata* were chosen for further evaluation at a lower concentration (50 μg/mL) against both H460 and A549 cell lines, as their inhibition percentages exceeded 60%. In these assays, both *N.annulata and V.brunneicolor* had higher cytotoxicity on H460 cells than A549 (Fig. 7b). Between species, *N.annulata* showed the highest inhibition of H460 cells (67.16%) and A549 cells (38.55%) compared to *V.brunneicolor*, which inhibited H460 and A549 cells by 57.33% and 23.64%, respectively (Fig. 7b). However, these differences were not statistically significant (p > 0.05). The IC_50_ values for both fungi were calculated as 70.71 μg/mL.

#### ﻿Antimicrobial assay

To assess the antifungal activity of *A.falcata* (CGMCC3.23740), *K.tumidula* (CGMCC3.23629), *N.annulata* (CGMCC3.23625), and *V.brunneicolor* (CGMCC3.23628), three fungal pathogens, *A.alternata*, *B.cinerea*, and *F.graminearum*, were used. *Amphisphaeriafalcata* (CGMCC3.23740) inhibited the growth of *F.graminearum* with an inhibition rate of 21%, but it also promoted the growth of *A.alternata* and *B.cinerea* (Fig. 7c, d). *Kirschsteiniotheliatumidula* (CGMCC3.23629) did not affect *A.alternata* but significantly promoted the growth of *B.cinerea* and *F.graminearum* (Fig. 7c, d). *Neoroussoellaannulata* (CGMCC3.23625) did not inhibit any of the three pathogens and instead promoted their growth (Fig. 7c, d). *Veronaeabrunneicolor* (CGMCC3.23628) inhibited all three pathogens, particularly *B.cinerea* and *F.graminearum*, with inhibition rates of 17% and 16%, respectively (Fig. 7c, d).

For the antibacterial assays, *Escherichiacoli* and *Staphylococcusaureus* were used as test organisms. None of the four fungi isolated in this study exhibited any antibacterial activity (Fig. 7e).

### ﻿Extracellular enzyme assay

**Figure 8.**
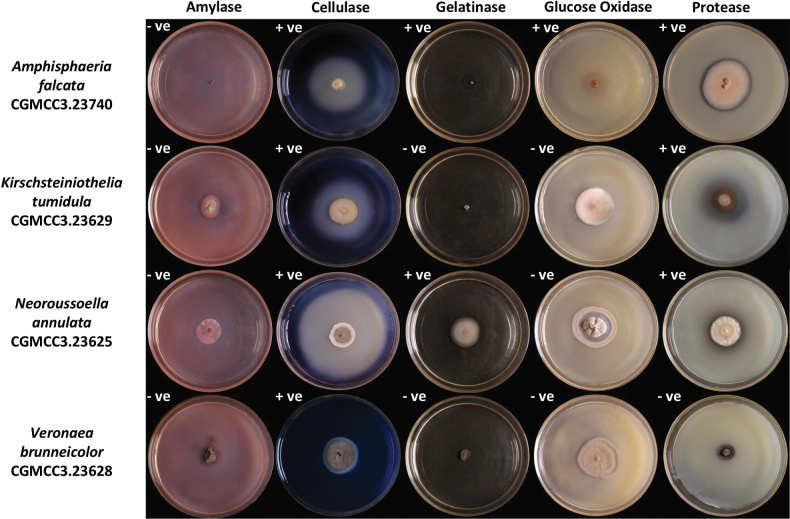
Extracellular enzymes assay of *Amphisphaeriafalcata* (CGMCC3.23740), *Kirschsteiniotheliatumidula* (CGMCC3.23629), *Neoroussoellaannulata* (CGMCC3.23625), and *Veronaeabrunneicolor* (CGMCC3.23628). Positive and negative reactions are denoted by the presence of a transparent circle, marked with a “+ve” and “-ve” symbol in the upper left corner of each plate, respectively. All experiments were performed in triplicate and repeated once.

The fungal strains exhibited diverse enzymatic profiles, emphasising their potential biocatalytic capabilities (Fig. 8). *Amphisphaeriafalcata* (CGMCC3.23740) displayed the widest enzymatic range, including cellulase, gelatinase, glucose oxidase, and protease activities. *Kirschsteiniotheliatumidula* (CGMCC3.23629) demonstrated both cellulase and protease activities. *Neoroussoellaannulata* (CGMCC3.23625) showed cellulase, gelatinase, and protease activities. Lastly, *Veronaeabrunneicolor* (CGMCC3.23628) exhibited cellulase activity.

## ﻿Discussion

In our previous study (Si et al. 2023), we successfully identified a range of endolichenic fungi from four species of *Usnea*. Initial classification of these fungi based on complete internal transcribed spacer (ITS) sequences revealed that some isolates shared limited similarities with known fungal species, indicating the potential presence of novel taxa. To explore this further, we expanded our investigation by amplifying and sequencing additional genetic markers, applying robust phylogenetic methods to formally describe these fungi as *Amphisphaeriafalcata*, *Kirschsteiniotheliatumidula*, *Neoroussoellaannulata*, and *Veronaeabrunneicolor*. Additionally, we assessed the biotechnological and pharmaceutical potential of these fungi by evaluating their anticancer, antimicrobial, and enzymatic activities. *Veronaeabrunneicolor* and *N.annulata* demonstrated moderate anticancer activity against H460 and A549 cell lines, significant inhibition of plant pathogenic fungi by *V.brunneicolor*, and the production of various enzymes, including cellulase, gelatinase, glucose oxidase, and protease, suggesting their potential for industrial applications.

This study introduces and describes four novel fungal species isolated from *Usnea* spp., significantly expanding the ecological range of their respective genera. Phylogenetic analyses utilising multiple gene regions identified these taxa within the genera *Amphisphaeria*, *Kirschsteiniothelia*, *Neoroussoella*, and *Veronaea*, each representing the first of its genus to be isolated from lichen. For instance, *Amphisphaeria* typically comprises species that are saprobic on woody branches and certain monocotyledons, with *Amphisphaeriaorixae* being the only known endophytic species before this study (Samarakoon et al. 2022; Wang et al. 2023; Li W-L et al. 2024). This discovery broadens the known ecological range of the genus. Similarly, *Kirschsteiniothelia* species, predominantly saprobes found on decaying wood in terrestrial and freshwater ecosystems (de Farias et al. 2024), and *Neoroussoella*, traditionally associated with decaying branches and plant materials (Liu et al. 2014; Dai et al. 2022; Hyde et al. 2023), are now represented by strains isolated from lichen. These findings significantly extend the current understanding of fungal ecology, highlighting the diverse fungal communities within lichen thalli and suggesting a more complex ecological network than previously recognised.

The four fungal species described in this study demonstrated varying degrees of cytotoxicity against the H460 and A549 cell lines, with *V.brunneicolor* and *N.annulata* showing moderate activity at a concentration of 100 μg/mL. However, *N.annulata* retained its cytotoxic potential at a lower concentration of 50 μg/mL, indicating a selectivity in its biological activity. This observation opens possibilities for targeted therapeutic applications, although further investigation is needed to fully elucidate the mechanisms behind this selectivity. In contrast, the remaining strains did not exhibit significant anticancer properties, highlighting the need for continued exploration of endolichenic fungi as a reservoir of bioactive compounds. Previous studies have revealed cytotoxic compounds in the *Veronaea* (Zhou et al. 2015), underscoring the promise of these fungi in drug discovery. Furthermore, the secondary metabolites of *A.orixae*, although not demonstrating significant antitumor activity (Wang et al. 2023), suggest that endolichenic fungi may possess yet-to-be-explored bioactive compounds.

In addition to their anticancer potential, the antimicrobial activity of the four strains was assessed. Despite the known antimicrobial properties of many endolichenic fungi (Kellogg and Raja 2017; Agrawal et al. 2020; Dumo et al. 2023), our study found no antibacterial activity against *S.aureus* and *E.coli* in any of the isolates. The antifungal activity was highly variable, with some species even promoting the growth of fungal pathogens. These results suggest that endolichenic fungi play complex and varied roles within lichen ecosystems, potentially contributing to the microbial balance of the lichen holobiont. This complexity highlights the necessity for further research to explore the ecological functions and potential applications of these fungi, as their antimicrobial effects may depend on environmental conditions or symbiotic relationships.

The extracellular enzyme assays revealed a remarkable diversity of enzymatic activities, including amylase, protease, gelatinase, glucose oxidase, and cellulase. By producing exoenzymes, microorganisms gain the capacity to metabolise diverse macromolecules for energy (Da Silva et al. 2022; Perera et al. 2022), which not only facilitates their colonisation of varied ecological niches but also drives key ecological processes, such as litter decomposition and the breakdown of senescent lichen thalli (Tripathi and Joshi 2019), through targeted enzymatic degradation of complex organic substrates. These enzymes are also of substantial biotechnological interest. For example, cellulase is widely used in the paper and textile industries, while protease is essential in the dairy sector. Glucose oxidase and amylase have applications in food preservation and biofuel production. Furthermore, the combination of these enzymes plays a critical role in environmental remediation by breaking down organic pollutants (Ghosh et al. 2023). The enzymatic capabilities of these endolichenic fungi suggest their potential for diverse industrial applications, ranging from waste management to bioremediation, and provide further justification for their investigation as biocatalysts.

## ﻿Conclusion

In conclusion, this study significantly expands the understanding of fungal diversity and functionality by identifying four novel fungal species isolated from lichen substrates. These species broaden the ecological spectrum of their genera and demonstrate a varied range of biological activities, including cytotoxicity, antimicrobial effects, and extracellular enzyme production. The diverse enzymatic capabilities and antimicrobial activities observed suggest that these fungi have multifaceted roles within the lichen ecosystem, which deserve further exploration. Collectively, these findings underscore the untapped biotechnological and pharmaceutical potential of endolichenic fungi, highlighting the need for continued research into their ecological roles and bioactive metabolites. The potential for novel bioactive compounds with implications for drug development, industrial applications, and environmental remediation is considerable, making these fungi an exciting area for future investigation.

## Supplementary Material

XML Treatment for Amphisphaeria falcata

XML Treatment for Kirschsteiniothelia tumidula

XML Treatment for Neoroussoella annulata

XML Treatment for Veronaea brunneicolor

## ﻿Additional information

### Author ORCIDs

Runlei Chang https://orcid.org/0000-0001-6613-9062

Zhaoqi Yan https://orcid.org/0009-0007-7188-2791

Jibo Jiang https://orcid.org/0009-0008-7072-8773

Yichen Wang https://orcid.org/0000-0001-6904-0594

Hongli Si https://orcid.org/0000-0003-0585-3478

Tanay Bose https://orcid.org/0000-0002-2069-042X

Congcong Miao https://orcid.org/0009-0003-6450-8145

### Data availability

All of the data that support the findings of this study are available in the main text or Supplementary Information.

All sequence data generated for this work can be accessed via GenBank: https://www.ncbi.nlm.nih.gov/genbank/.

## Supplementary materials

Supplementary material 1 Supplementary tables Data type docx Explanation note **table S1.** List of fungal primers used for amplification of various gene regions for the identification of endolichenic fungi isolated from various species of *Usnea*. **table S2.** List of *Amphisphaeria* species used for the phylogenetic study. *Bartaliniapini* and *Bartaliniapondoensis* served as the outgroup. Taxon names with the suffix “T” denote ex-type cultures. Newly generated sequences are shown in bold. **table S3.** List of *Kirschsteiniothelia* species used for the phylogenetic study. *Acrospermumadeanum* and *Acrospermumcompressum* served as the outgroup. Taxon names with the suffix “T” denote ex-type cultures. Newly generated sequences are shown in bold. **table S4.** List of *Veronaea* and related species used for the phylogenetic study. *Cyphellophoraoxyspora* served as the outgroup. Taxon names with the suffix “T” denote ex-type cultures. Newly generated sequences are shown in bold. **table S5.** List of Thyridariaceae species used for the phylogenetic study. *Torulaherbarum* and *Torulahollandica* served as the outgroup. Taxon names with the suffix “T” denote ex-type cultures. Newly generated sequences are shown in bold. https://binary.pensoft.net/file/1343198This dataset is made available under the Open Database License (http://opendatacommons.org/licenses/odbl/1.0/). The Open Database License (ODbL) is a license agreement intended to allow users to freely share, modify, and use this Dataset while maintaining this same freedom for others, provided that the original source and author(s) are credited.Runlei Chang, Zhaoqi Yan, Jibo Jiang, Yichen Wang, Hongli Si, Tanay Bose, Congcong Miao
