# Supplementary material for: ﻿Four novel endolichenic fungi from Usnea spp. (Lecanorales, Parmeliaceae) in Yunnan and Guizhou, China: Taxonomic description and preliminary assessment of bioactive potentials
Source: MycoKeys. 2025 Jun 2;118:55–80. doi: 10.3897/mycokeys.118.155248 (PMC12149887; doi:10.3897/mycokeys.118.155248)
Supplement: Supplementary material 1 — Supplementary tables [file mycokeys-118-055-s001.docx]

**Supplementary Tables**

**Table S1** List of fungal primers used for amplification of various gene regions for the identification of endolichenic fungi isolated from various species of *Usnea*.

| **Gene regions** | | **Primers** | **Annealing temperature** | | **References** |
| --- | --- | --- | --- | --- | --- |
| TEF | EF-986R | | 55 °C | (Carbone and Kohn 1999; Groenewald et al. 2013) | |
|  | EF-983F | |  |  | |
|  | EF1-728F | |  |  | |
|  | EF-TEF1LLErev | |  |  | |
|  | EF-983F | |  |  | |
|  | EF-2218R | |  |  | |
| RPB2 | fRPB2-5F | | 55 °C | (Liu et al. 1999) | |
|  | fRPB2-7cR | |  |  | |
| LSU | LR5 | | 55 °C | (Vilgalys and Hester 1990; White et al. 1990) | |
|  | LR0R | |  |  | |
| SSU | NS1 | | 55 °C | (White et al. 1990) | |
|  | NS4 | |  |  | |
| TUB | Bt2a | | 55 °C | (O"Donnell and Cigelnik 1997) | |
|  | Bt2b | |  |  | |
|  | T2 | |  |  | |
|  | T10 | |  |  | |

**References**

Carbone I, Kohn LM. 1999. A method for designing primer sets for speciation studies in filamentous ascomycetes. Mycologia. 91(3):553–556. doi: 10.1080/00275514.1999.12061051

Groenewald JZ, Nakashima C, Nishikawa J, Shin HD, Park JH, Jama AN, Groenewald M, Braun U, Crous PW. 2013. Species concepts in *Cercospora*: spotting the weeds among the roses. Stud. Mycol. 75(1):115–170. doi: 10.3114/sim0012

Liu YJ, Whelen S, Hall BD. 1999. Phylogenetic relationships among ascomycetes: evidence from an RNA polymerse II subunit. Mol. Biol. Evol. 16(12):1799–1808. doi: 10.1093/oxfordjournals.molbev.a026092

O"Donnell K, Cigelnik E. 1997. Two divergent intragenomic rDNA ITS2 types within a monophyletic lineage of the fungus Fusarium are nonorthologous. Mol. Phylogenet. Evol. 7(1):103–116. doi: 10.1006/mpev.1996.0376

Vilgalys R, Hester M. 1990. Rapid genetic identification and mapping of enzymatically amplified ribosomal DNA from several species of *Cryptococcus*. J. Bacteriol. 172:4238–4246. doi: 10.1128/jb.172.8.4238-4246.1990

White TJ, Bruns T, Lee S, J Ta. 1990. Amplification and Direct Sequencing of Fungal Ribosomal RNA Genes for Phylogenetics. In: Innis MA, Gelfand DH, Sninsky JJ, White TJ (Eds) PCR Protocols Academic Press, San Diego.315–322.

**Table S2** List of *Amphisphaeria* species used for phylogenetic study. *Bartalinia pini* and *Bartalinia pondoensis* served as the outgroup. Taxon name with a suffixed with T indicates ex-type culture. Newly generated sequences are indicated in bold.

| **Species** | **Isolate** | **GenBank numbers** | | |  |
| --- | --- | --- | --- | --- | --- |
|  |  | **LSU** | **ITS** | **RPB2** | **BT** |
| *Amphisphaeria ailaoshanensis* | KUNCC 23-15520^T^ | PP584770 | PP584673 | - | - |
| *Amphisphaeria ailaoshanensis* | KUNCC 23-15521 | PP584771 | PP584674 | - | - |
| *Amphisphaeria acericola* | MFLU 16-2479 ^T^ | MK640424 | MK640423 | - | - |
| *Amphisphaeria camelliae* | HKAS 107021^T^ | MT756615 | MT756621 | MT789850 | MT774368 |
| *Amphisphaeria camelliae* | MFLUCC 20-0122 | MT756616 | MT756622 | MT789851 | MT774369 |
| *Amphisphaeria chiangmaiensis* | CMUB 40017 | OR507152 | OR507139 | OR504416 | - |
| *Amphisphaeria chiangmaiensis* | MFLU 23-0411^T^ | OR507153 | OR507140 | - | - |
| *Amphisphaeria curvaticonidia* | HKAS 102288 | MT756618 | MT756624 | MT789853 | - |
| *Amphisphaeria curvaticonidia* | MFLUCC 18-0620^T^ | MT756617 | MT756623 | MT789852 | - |
| ***Amphisphaeria falcata*** | **CGMCC3.23740^T^** | **OQ645284** | **OQ645270** | **OQ696281** | **OQ696283** |
| *Amphisphaeria flava* | MFLUCC 18-0361^T^ | MH971234 | MH971224 | - | MK033638 |
| *Amphisphaeria fuckelii* | WU 33555 | KT949903 | KT949903 | - | - |
| *Amphisphaeria fuckelii* | CBS 140409^T^ | KT949902 | KT949902 | MH554918 | MH554677 |
| *Amphisphaeria hydei* | CMUB 40016 | OR507154 | OR507141 | OR504417 | OR519975 |
| *Amphisphaeria hydei* | MFLU 23-0412^T^ | OR507155 | OR507142 | OR504418 | OR519976 |
| *Amphisphaeria karsti* | GZAAS 20-0147T | OR209622 | OR224991 | - | - |
| *Amphisphaeria karsti* | GZAAS 20-0148 | OR209623 | OR224992 | - | - |
| *Amphisphaeria kunmingensis* | KUNCC 23-15522^T^ | PP584772 | PP584675 | - | - |
| *Amphisphaeria kunmingensis* | KUNCC 23-15523 | PP584773 | PP584676 | - | - |
| *Amphisphaeria magna* | HKAS 130270^T^ | PP584774 | PP584677 | - | - |
| *Amphisphaeria magna* | HKAS 130271 | PP584775 | PP584678 | - | - |
| *Amphisphaeria mangrovei* | NFCCI-4247^T^ | MG844275 | MG844283 | - | - |
| *Amphisphaeria micheliae* | HKAS 107012^T^ | MT756619 | MT756625 | MT789854 | MT774370 |
| *Amphisphaeria micheliae* | MFLUCC 20-0121 | MT756620 | MT756626 | MT789855 | MT774371 |
| *Amphisphaeria neoaquatica* | MFLUCC 14-0045^T^ | MK835805 | MK828607 | - | - |
| *Amphisphaeria oleae* | CGMCC 3.24959^T^ | OR253313 | OR253156 | OR253756 | OR266102 |
| *Amphisphaeria oleae* | UESTCC 23.0120 | OR253314 | OR253157 | OR253757 | OR266103 |
| *Amphisphaeria orixae* | GZCC 22-2031^T^ | OQ064543 | OQ064541 | - | - |
| *Amphisphaeria orixae* | GZCC 22-2032 | OQ064544 | OQ064542 | - | - |
| *Amphisphaeria parvispora* | MFLU 18-0767 ^T^ | NG_081501 | NR_175677 | MW658631 | MW775601 |
| *Amphisphaeria qujingensis* | KUMCC 19-0187^T^ | MN556316 | MN477033 | - | - |
| *Amphisphaeria qujingensis* | KUMCC 19-0186 | MN707566 | MN707568 | - | - |
| *Amphisphaeria sambuci* | CBS 131707^T^ | KT949904 | KT949904 | MH554911 | MH704632 |
| *Amphisphaeria sambuci* | WU 33557 | KT949905 | KT949905 | - | - |
| *Amphisphaeria shangrilaensis* | HKAS 130272^T^ | PP584776 | PP584679 | - | - |
| *Amphisphaeria shangrilaensis* | HKAS 130273 | PP584777 | PP584680 | - | - |
| *Amphisphaeria sorbi* | MFLUCC 13-0721^T^ | KP744475 | KR092797 | - | - |
| *Amphisphaeria thailandica* | MFLU 18-0794^T^ | MH971235 | MH971225 | MK033640 | MK033639 |
| *Amphisphaeria umbrina* | AFTOL-ID 1229 | FJ176863 | - | - | - |
| *Amphisphaeria uniseptata* | CBS 114967^T^ | MH554197 | - | MH554878 | MH554638 |
| *Amphisphaeria verniciae* | CGMCC 3.24960^T^ | OR253269 | OR253154 | OR251139 | OR266100 |
| *Amphisphaeria verniciae* | UESTCC 23.0122 | OR253270 | OR253155 | OR251140 | OR266101 |
| *Amphisphaeria xishuangbannaense* | KUNCC 23-15524^T^ | PP584778 | PP584681 | - | - |
| *Amphisphaeria xishuangbannaense* | KUNCC 23-15525 | PP584779 | PP584682 | - | - |
| *Amphisphaeria yunnanensis* | KUMCC 19-0188^T^ | MN556306 | MN477177 | - | - |
| *Amphisphaeria yunnanensis* | KUMCC 19-0189 | MN550992 | MN550997 | - | - |
| *Bartalinia pini* | CBS 143891^T^ | MH554330 | MH554125 | MH555033 | MH554797 |
| *Bartalinia pondoensis* | CBS 125525^T^ | MH875078 | MH863602 | MH554904 | MH554663 |

**Table S3** List of *Kirschsteiniothelia* species used for phylogenetic study. *Acrospermum adeanum* and *Acrospermum compressum* served as the outgroup. Taxon name with a suffixed with T indicates ex-type culture. Newly generated sequences are indicated in bold.

| **Species** | **Isolate** | **GenBank numbers** | | |
| --- | --- | --- | --- | --- |
|  |  | **ITS** | **LSU** | **SSU** |
| *Acrospermum adeanum* | M133 | EU940180 | EU940104 | EU940031 |
| *Acrospermum compressum* | M151 | EU940161 | EU940084 | EU940012 |
| *Kirschsteiniothelia acutispora* | MFLU 21-0127^T^ | OP120780 | ON980758 | ON980754 |
| *Kirschsteiniothelia aethiops* | CBS 109.53 | - | AY016361 | AY016344 |
| *Kirschsteiniothelia aethiops* | MFLUCC 15-0424 | KU500571 | KU500578 | KU500585 |
| *Kirschsteiniothelia agumbensis* | NFCCI 5714 ^T^ | PP029048 | - | PP029049 |
| *Kirschsteiniothelia aquatica* | MFLUCC 17–1685^T^ | MH182587 | MH182594 | MH182618 |
| *Kirschsteiniothelia arasbaranica* | IRAN 2508C^T^ | KX621983 | KX621984 | KX621985 |
| *Kirschsteiniothelia arasbaranica* | IRAN 2509C | KX621986 | KX621987 | KX621988 |
| *Kirschsteiniothelia atra* | DEN | MG602687 | - | - |
| *Kirschsteiniothelia bulbosapicalis* | GZCC 23-0732 ^T^ | PQ248937 | PQ248933 | PQ248929 |
| *Kirschsteiniothelia cangshanensis* | MFLUCC 16-1350^T^ | MH182584 | MH182592 | - |
| *Kirschsteiniothelia cangshanensis* | GZCC19-0515 | - | MW133829 | MW134609 |
| *Kirschsteiniothelia chiangmaiensis* | MFLU 23-0358^T^ | NR_190974 | NG_243391 | NG_243004 |
| *Kirschsteiniothelia crustaceum* | T20-1127^T^ | MW851849 | MW851854 |  |
| *Kirschsteiniothelia dendryphioides* | KUNCC 10431 ^T^ | OP626354 | PQ248935 | PQ248931 |
| *Kirschsteiniothelia dendryphioides* | KUNCC 10499 | PQ248938 | - | - |
| *Kirschsteiniothelia dushanensis* | GZCC:19-0415^T^ | OP377845 | - | - |
| *Kirschsteiniothelia ebriosa* | FMR:16666^T^ | - | LT985884 | - |
| *Kirschsteiniothelia ebriosa* | FMR:16665 | - | LT985885 | - |
| *Kirschsteiniothelia emarceis* | MFLUCC 10-0037^T^ | NR_138375 | NG_059454 | HQ441572 |
| *Kirschsteiniothelia esperanzae* | T. Raymundo 6581 ^T^ | OQ877253 | OQ880482 | - |
| *Kirschsteiniothelia extensum* | MFLU 21-0130/T20-1129 ^T^ | MW851850 | MW851855 | - |
| *Kirschsteiniothelia fluminicola* | MFLUCC 16-1263^T^ | MH182582 | MH182588 | - |
| *Kirschsteiniothelia ganzhouensis* | HJAUP C1209 ^T^ | PP505546 | PP506568 | PP527763 |
| *Kirschsteiniothelia ganzhouensis* | HJAUP C1210 | PQ456024 | PQ443751 | PQ443763 |
| *Kirschsteiniothelia guangdongensis* | MHZU:22-0137^T^ | - | OR164974 | - |
| *Kirschsteiniothelia guizhouensis* | GZCC 24-0034^T^ | PQ404852 | PQ404856 | PQ404859 |
| *Kirschsteiniothelia guizhouensis* | GZCC 24-0041 | PQ404853 | - | PQ404860 |
| *Kirschsteiniothelia inthanonensis* | MFLUCC 23-0277 ^T^ | OR762773 | OR762781 | OR764784 |
| *Kirschsteiniothelia jiangxiensis* | HJAUP C1273 ^T^ | PP505548 | PP506566 | PP506565 |
| *Kirschsteiniothelia jiangxiensis* | HJAUP C1274 | PQ456026 | PQ443753 | PQ443765 |
| *Kirschsteiniothelia jiulianshanensis* | HJAUP C1313 ^T^ | PP505549 | PP506562 | PP506563 |
| *Kirschsteiniothelia jiulianshanensis* | HJAUP C1314 | PQ456028 | PQ443755 | PQ443767 |
| *Kirschsteiniothelia laojunensis* | KUN-L 88727^T^ | PP081651 | PP081658 | - |
| *Kirschsteiniothelia lignicola* | MFLUCC 10-0036^T^ | HQ441567 | HQ441568 | HQ441569 |
| *Kirschsteiniothelia longirostrata* | GZCC 23-0733 ^T^ | PQ248939 | PQ248934 | PQ248930 |
| *Kirschsteiniothelia longisporum* | UESTCC 24.0190 ^T^ | PQ038266 | PQ038273 | PQ046108 |
| *Kirschsteiniothelia nabanheensis* | HJAUP C2004^T^ | OQ023197 | OQ023273 | OQ023038 |
| *Kirschsteiniothelia nabanheensis* | HJAUP C2006 | OQ023274 | OQ023275 | OQ023037 |
| *Kirschsteiniothelia phoenicis* | MFLUCC 18-0216^T^ | MG859978 | MG860484 | MG859979 |
| *Kirschsteiniothelia pini* | UESTCC 24.0131^T^ | PP835321 | PP835315 | PP835318 |
| *Kirschsteiniothelia puerensis* | ZHKUCC:22-0271^T^ | OP450977 | OP451017 | OP451020 |
| *Kirschsteiniothelia puerensis* | ZHKUCC:22-0272 | OP450978 | OP451018 | OP451021 |
| *Kirschsteiniothelia ramus* | GZCC 23-0596 ^T^ | OR098711 | OR091333 | - |
| *Kirschsteiniothelia rostrata* | MFLUCC 15-0619^T^ | KY697280 | KY697276 | KY697278 |
| *Kirschsteiniothelia rostrata* | MFLUCC 16-1124 | - | MH182590 | - |
| *Kirschsteiniothelia saprophytica* | MFLUCC 23-0275^T^ | OR762774 | OR762783 | - |
| *Kirschsteiniothelia saprophytica* | MFLUCC 23-0276 | OR762775 | OR762782 | - |
| *Kirschsteiniothelia septemseptatum* | MFLU 21-0126^T^ | NR_185750 | ON980757 | ON980752 |
| *Kirschsteiniothelia sichuanensis* | UESTCC 24.0127^T^ | PP785368 | PP784322 | - |
| *Kirschsteiniothelia spatiosum* | MFLU 21-0128^T^ | NR_187065 | - | ON980753 |
| *Kirschsteiniothelia submersa* | MFLUCC 15-0427^T^ | KU500570 | KU500577 | KU500584 |
| *Kirschsteiniothelia submersa* | S-481 | - | MH182591 | MH182616 |
| *Kirschsteiniothelia tectonae* | MFLUCC 12-0050^T^ | KU144916 | KU764707 | - |
| *Kirschsteiniothelia thailandica* | MFLUCC 20-0116^T^ | MT985633 | MT984443 | MT984280 |
| *Kirschsteiniothelia thujina* | JF 13210 | KM982716 | KM982718 | KM982717 |
| ***Kirschsteiniothelia tumidula*** | **CGMCC3.23629^T^** | **OQ645272** | **OQ645286** | **OQ645279** |
| *Kirschsteiniothelia vinigena* | FMR 15668^T^ | - | NG_075229 | - |
| *Kirschsteiniothelia weiningensis* | GZCC 24-0072^T^ | PQ404851 | PQ404855 | PQ404858 |
| *Kirschsteiniothelia xishuangbannaensis* | ZHKUCC 22-0220^T^ | OP289566 | OP289564 | OP303181 |
| *Kirschsteiniothelia xishuangbannaensis* | ZHKUCC 22-0221 | OP289563 | OP289565 | OP303182 |
| *Kirschsteiniothelia xishuiensis* | GZCC 24-0052^T^ | PQ404850 | PQ404854 | PQ404857 |
| *Kirschsteiniothelia zizyphifolii* | MFLUCC 23-027^T^ | OR762768 | OR762776 | OR764779 |

**Table S4** List of *Veronaea* and related species used for phylogenetic study. *Cyphellophora oxyspora* served as the outgroup. Taxon name with a suffixed with T indicates ex-type culture. Newly generated sequences are indicated in bold.

| **Species** | **Isolate** | **GenBank numbers** | | | | |
| --- | --- | --- | --- | --- | --- | --- |
|  |  | **LSU** | **SSU** | **ITS** | **BT** | **TEF** |
| *Aculeata aquatica* | MFLUCC 11-0529 T | MG922575 | MG922579 | MG922571 | - | - |
| *Brycekendrickomyces acaciae* | CBS 124104 T | FJ839641 | - | FJ839606 | - | - |
| *Capronia pilosella* | AFTOL-ID 657 | DQ823099 | DQ823106 | - | - | - |
| *Cladophialophora carrionii* | CBS 160.54 T | FJ358234 | FJ358302 | AF050262 | - | - |
| *Cladophialophora minourae* | CBS 556.83 T | FJ358235 | FJ358303 | AY251087 | - | EU140598 |
| *Cladophialophora parmeliae* | CBS 129337 | JQ342182 | - | JQ342180 | - | - |
| *Cyphellophora oxyspora* | CBS 698.73 T | KC455262 | KC455305 | KC455249 | KC455232 | - |
| *Exophiala aquamarina* | CBS 119918 T | - | JN856012 | JF747054 | JN112434 | XM_013405206 |
| *Exophiala equina* | CBS 119.23 T | - | JN856017 | NR_111627 | JN112462 | - |
| *Exophiala jeanselmei* | CBS 507.90 T | NG_070514 | NG_062765 | NR_111129 | EF551501 | EF551530 |
| *Exophiala pisciphila* | CBS 537.73 T | MH872483 | NG_013192 | NR_121269 | JN112493 | JN128788 |
| *Exophiala psychrophila* | CBS 191.87 T | MH873750 | JN856019 | JF747135 | JN112497 | JN128798 |
| *Exophiala nagquensis* | CGMCC 3.17333 T | KP347960 | NG_074893 | NR_172237 | KP347924 | KP347914 |
| *Exophiala nagquensis* | CGMCC 3.17334 | MG197837 | MG012741 | KP347949 | KP347923 | KP347915 |
| *Exophiala salmonis* | CBS 157.67 T | MH870616 | JN856020 | JF747137 | JN112499 | JN128747 |
| *Exophiala tremulae* | CBS 129355 T | - | NG_063060 | NR_159874 | KT894148 | KT894149 |
| *Fonsecaea erecta* | CBS 125763 T | KF155186 | KF155210 | KC886414 | KF155221 | KF155227 |
| *Fonsecaea monophora* | CBS 102243 | FJ358247 | FJ358315 | EU938579 | EU938542 | - |
| *Marinophialophora garethjonesii* | KUMCC 16-0066 T | KY305177 | KY305179 | KY305175 | - | - |
| *Melanoctona tectonae* | MFLUCC 12-0389 T | KX258779 | KX258780 | KX258778 | - | - |
| *Metulocladosporiella musae* | CBS 161.74 T | DQ008161 | - | DQ008137 | - | MG934478 |
| *Minimelanolocus aquaticus* | MFLUCC 15-0414 T | NG_070385 | NG_063577 | NR_154181 | - | - |
| *Minimelanolocus asiaticus* | MFLUCC 15-0237 T | NG_070384 | NG_063576 | NR_154179 |  | - |
| *Minimelanolocus clavatus* | DLUCC 3022 T | MT271772 | MT271777 | MT271774 | - | - |
| *Minimelanolocus submersus* | KUMCC 15-0206 T | KX789215 | - | KX789212 | - | - |
| *Phialophora verrucosa* | CBS 140325 T | - | NG_061187 | NR_146242 | - | - |
| *Ramichloridium anceps* | CBS 181.65 T | NG_027584 | NG_013193 | EU041805 | - | DQ840568 |
| *Thysanorea aquatica* | MFLUCC 15-0966 T | MG922576 | MG922580 | MG922572 | - | - |
| *Thysanorea papuana* | CBS 212.96 T | EU041871 | - | EU041814 | - | - |
| *Veronaea aquatica* | JAUCC2549 T | MW046893 | - | MW046892 | MW248394 | - |
| *Veronaea botryosa* | CBS 254.57 T | EU041873 | NG_061120 | EU041816 | JN112505 | - |
| *Veronaea botryosa* | CBS 102593 | KF928493 | - | KF928429 | KF928557 | - |
| ***Veronaea brunneicolor*** | **CGMCC3.23628** | **OQ645285** | **OQ645278** | **OQ645271** | **OQ696284** | - |
| *Veronaea compacta* | CBS 268.75 T | EU041876 | - | EU041819 | - | - |
| *Veronaea japonica* | CBS 776.83 T | EU041875 | - | EU041818 | - | - |
| *Veronaea polyconidia* | CGMCC 3.25589 T | OR807868 | OR807865 | OR807862 | OR817660 | - |
| *Veronaea polyconidia* | UESTCC 23.0138 | OR807869 | OR807866 | OR807863 | OR817661 | - |

**Table S5** List of Thyridariaceae species used for phylogenetic study. *Torula herbarum* and *Torula hollandica* served as the outgroup. Taxon name with a suffixed with T indicates ex-type culture. Newly generated sequences are indicated in bold.

| **Species** | **Isolate** | **Genbank numbers** | | | | |
| --- | --- | --- | --- | --- | --- | --- |
|  |  | **ITS** | **LSU** | **SSU** | **TEF** | **RPB2** |
| *Chromolaenomyces appendiculatus* | MFLUCC_17-1455^T^ | NR_168862 | NG_068705 | MT214394 | MT235770 | MT235806 |
| *Cycasicola goaensis* | MFLU_17-058 ^T^ | NR_157510 | NG_059057 | NG_061287 | MG829198 | - |
| *Cycasicola leucaenae* | MFLUCC 17-0914^T^ | NR_163322 | NG_070071 | NG_065771 | MK360046 | MK434900 |
| *Elongatopedicellata aquatica* | FMR 17834^T^ | - | NG_149053 | - | - | - |
| *Elongatopedicellata lignicola* | MFLUCC:15-0642^T^ | - | KX421368 | KX421369 | - | - |
| *Liua muriformis* | KUMCC_18-0177^T^ | NR_164301 | NG_066444 | NG_067706 | MK426798 | MK426799 |
| *Neoroussoella alishanense* | FU31016 ^T^ | MK503816 | MK503822 | MK503828 | MK336181 | MN037756 |
| ***Neoroussoella annulata*** | **CGMCC3.23625^T^** | **OQ645267** | **OQ645281** | - | **OQ696275** | **OQ696278** |
| *Neoroussoella bambusae* | MFLUCC 11-0124 ^T^ | KJ474827 | KJ474839 | - | KJ474848 | KJ474856 |
| *Neoroussoella chiangmaiensis* | MFLU 22-0205^T^ | OQ065738 | OQ065735 | OQ065736 | OQ186448 | OQ186450 |
| *Neoroussoella clematidis* | MFLU 17-1467^T^ | NR_170813 | MT214587 | MT226700 | MT394645 | MT394701 |
| *Neoroussoella entadae* | MFLUCC 17-0920^T^ | NR_163325 | - | NG_065773 | - | MK434898 |
| *Neoroussoella fulvicomae* | MFLU 17-1471^T^ | NR_170814 | MT214588 | NG_070664 | MT394646 | MT394702 |
| *Neoroussoella heveae* | MFLUCC 17-1983 ^T^ | MH590693 | MH590689 | MH590691 | - | - |
| *Neoroussoella lenispora* | GZCC 16-0020 ^T^ | - | KX791431 | - | - | - |
| *Neoroussoella leucaenae* | MFLUCC 18-1544 ^T^ | MK347767 | MK347984 | NG_065774 | MK360067 | MK434876 |
| *Neoroussoella lignicola* | MUT 5373^T^ | NR_169908 | MN556321 | KU314954 | MN605896 | MN605916 |
| *Neoroussoella magnoliae* | MFLU 18-1022^T^ | MK801232 | MK801230 | MK801231 | MK834373 | - |
| *Neoroussoella peltophora* | MFLUCC 21-0071^T^ | MZ567051 | NG_149003 | NG_148873 | - | - |
| *Neoroussoella sedimenticola* | CGMCC 3.22468^T^ | OQ798948 | OQ758143 | - | OQ809045 | OQ809007 |
| *Neoroussoella solani* | CPC 26331 ^T^ | KX228261 | KX228312 | - | - | - |
| *Neoroussoella thailandica* | NI258^T^ | - | - | - | - | ON502386 |
| *Nothoroussoella irregularis* | CGMCC 3.22466^T^ | OQ798955 | OQ758150 | OQ758183 | OQ809052 | OQ809014 |
| *Pararoussoella juglandicola* | CBS145037^T^ | MK442607 | MK442543 | - | MK442699 | MK442671 |
| *Pararoussoella mukdahanensis* | MFLUCC 11-0201 ^T^ | KU940129 | KU863118 | KU872121 | - | - |
| *Pararoussoella quercina* | CPC 34864 | NR_170060 | MT223920 | - | - | - |
| *Pararoussoella rosarum* | MFLUCC 17-0796 ^T^ | NR_157529 | NG059872 | NG_061294 | MG829224 | - |
| *Parathyridaria clematidis* | MFLUCC 17-2185^T^ | MT310642 | MT214598 | NG_070668 | MT394654 | MT394709 |
| *Parathyridaria ellipsoidea* | KNU-JJ-1829^T^ | LC552950 | LC552952 |  |  |  |
| *Parathyridaria ephedrae* | MCC 9655^T^ | NR_182700 | NG_154042 | - | - | - |
| *Parathyridaria flabelliae* | MUT 4859^T^ | KR014355 | KP671716 | NG_070285 | MN605909 | MN605929 |
| *Parathyridaria percutanea* | CBS 868.95 ^T^ | KF322118 | KF366449 | NG_062999 | KF407987 | KF366452 |
| *Parathyridaria philadelphi* | CBS:143432^T^ | MH107905 | NG_063958 | - | MH108023 | - |
| *Parathyridaria ramulicola* | CBS 141479 ^T^ | KX650565 | KX650565 | NG_061254 | KX650536 | KX650584 |
| *Parathyridaria robiniae* | MFLUCC 14-1119 ^T^ | KY511142 | KY511141 | - | KY549682 | - |
| *Parathyridaria rosae* | MFLU 17-0623^T^ | NR_157530 | NG_059873 | - | - | - |
| *Parathyridaria serratifoliae* | MFLUCC 17-2210^T^ | MT310646 | MT214602 | NG_070669 | MT394658 | MT394713 |
| *Parathyridaria tyrrhenica* | MUT 5371^T^ | NR_169907 | NG_075309 | NG_070291 | MN605912 | MN605932 |
| *Parathyridaria virginianae* | MFLUCC 17-2163^T^ | MT310647 | NG_073853 | NG_070670 | MT394659 | MT394714 |
| *Parathyridariella dematiacea* | MUT 4884^T^ | NR_169701 | KP671726 | NG_070286 | MN605906 | MN605926 |
| *Pseudoneoconiothyrium rosae* | MFLUCC 15-0052 ^T^ | NR_157523 | NG059868 | NG_063686 | - | - |
| *Pseudoroussoella chromolaenae* | MFLUCC 17-1492 ^T^ | NR_168861 | NG_068704 | NG_070154 | MT235769 | - |
| *Pseudoroussoella elaeicola* | MFLUCC 17-1483 | MT214348 | MT214442 | MT214396 | MT235772 | MT235808 |
| *Pseudothyridariella chromolaenae* | MFLUCC 17-1472^T^ | NR_168863 | NG_068706 | MT214395 | MT235771 | MT235807 |
| *Pseudothyridariella idesiae* | CGMCC 3.24439^T^ | NR_190270 | OR253307 | OR253216 | OR251154 | OR253762 |
| *Pseudothyridariella mahakashae* | PUFD99 | MG020435 | MG020438 | MG020441 | MG023140 | MG020446 |
| *Roussoella angustior* | MFLU 15-1214^T^ | - | NG_059587 | - | - | - |
| *Roussoella aquatica* | MFLUCC 18-1040 ^T^ | NR171975 | NG073797 | NG_073545 | - | - |
| *Roussoella arundinacea* | CBS 146088 ^T^ | MT223838 | MT223928 | - | MT223723 | - |
| *Roussoella bambusarum* | GMBC0316^T^ | ON479891 | ON479892 | - | - | - |
| *Roussoella chiangraina* | MFLUCC 10-0556^T^ | NR_155712 | NG_059510 | - | KJ474849 | KJ474857 |
| *Roussoella chinensis* | KUNCC 22-12534 | OP555451 | OP555449 | - | - | - |
| *Roussoella doimaesalongensis* | MFLUCC 14-0584^T^ | KY026584 | KY000659 | - | KY651249 | KY678394 |
| *Roussoella euonymi* | CBS 143426^T^ | NR_172280 | MH107961 | - | - | MH108007 |
| *Roussoella fusispora* | UESTCC 23.0135 | OR141720 | OR142438 |  | OR161835 | OR161837 |
| *Roussoella guttulata* | MFLUCC 20-0102^T^ | NR_172428 | NG_075383 | - | MW022188 | MW022187 |
| *Roussoella hysterioides* | CBS 546.94 | KF443405 | KF443381 | AY642528 | KF443399 | KF443392 |
| *Roussoella intermedia* | CBS 170.96 | KF443407 | KF443382 | KF443390 | KF443398 | KF443394 |
| *Roussoella japanensis* | MAFF 239636^T^ | NR_155713 | AB524621 | AB524480 | AB539114 | AB539101 |
| *Roussoella kunmingensis* | KUMCC 18-0128^T^ | MH453491 | MH453487 | - | MH453480 | MH453484 |
| *Roussoella magnatum* | MFLUCC 15-0185^T^ | - | KT281980 | - | - | - |
| *Roussoella mangrovei* | MFLUCC 16-0424^T^ | MH025951 | MH023318 | - | MH028246 | MH028250 |
| *Roussoella margidorensis* | MUT 5329^T^ | KU314944 | MN556322 | NG_070316 | MN605897 | MN605917 |
| *Roussoella mediterranea* | MUT 5369^T^ | KU314947 | MN556324 | NG_070290 | MN605899 | MN605919 |
| *Roussoella mexicana* | CPC 25355^T^ | KT950848 | KT950862 | - | - | - |
| *Roussoella multiloculate* | GMBCC1056^T^ | OM891799 | OM884015 | OM891821 | ON098343 | ON098369 |
| *Roussoella neopustulans* | MFLUCC 11-0609^T^ | KJ474833 | KJ474841 | - | KJ474850 | - |
| *Roussoella nitidula* | MFLUCC 11-0182^T^ | KJ474835 | KJ474843 | - | KJ474852 | KJ474859 |
| *Roussoella padinae* | MUT 5503^T^ | KU158170 | MN556327 | NG_070317 | MN605902 | MN605922 |
| *Roussoella papillate* | GMBCC1121^T^ | OM891814 | OM755608 | - | ON098346 | ON098378 |
| *Roussoella pseudohysterioides* | MFLUCC 13-0852^T^ | KU940131 | KU863120 | NG_065084 | KU940198 | - |
| *Roussoella pustulans* | MAFF 239637^T^ | KJ474830 | AB524623 | AB524482 | AB539116 | AB539103 |
| *Roussoella scabrispora* | MFLUCC 11-0624^T^ | KJ474836 | KJ474844 | - | KJ474853 | KJ474860 |
| *Roussoella siamensis* | MFLUCC 11-0149^T^ | KJ474837 | KJ474845 | NG_065086 | KJ474854 | KJ474861 |
| *Roussoella sichuanensis* | UESTCC 23.0136 | OR141721 | OR142439 |  | OR161836 | OR161838 |
| *Roussoella sinensis* | GMBCC1119^T^ | OM891813 | OM884024 | OM891833 | ON098357 | ON098379 |
| *Roussoella thailandica* | MFLUCC 11-0621^T^ | KJ474838 | KJ474846 | - | - | - |
| *Roussoella tuberculata* | MFLUCC 13-0854^T^ | KU940132 | KU863121 | NG_065085 | KU940199 | - |
| *Roussoella uniloculata* | GMBCC1110^T^ | OM891809 | OM801286 | OM891829 | ON098360 | ON098374 |
| *Roussoella verrucispora* | CBS 125434^T^ | KJ474832 | AB524622 | AB524481 | AB539115 | AB539102 |
| *Roussoella yunnanensis* | KUMCC 18-0115^T^ | MH453492 | MH453488 | - | MH453481 | - |
| *Roussoellopsis tosaensis* | KT 1659^T^ | - | AB524625 |  | AB539117 | AB539104 |
| *Setoarthopyrenia chromolaenae* | MFLUCC 17-1444^T^ | MT214344 | MT214438 | NG_070153 | MT235768 | MT235805 |
| *Thyridaria acaciae* | CBS 138873^T^ | KP004469 | KP004497 | - | - | - |
| *Thyridaria aureobrunnea* | MFLUCC 21-0090^T^ | NR_182937 | NG_088276 | - | - | - |
| *Thyridaria broussonetiae* | CBS 141481^T^ | NR_147658 | KX650568 | NG_063067 | KX650539 | KX650586 |
| *Thyridaria jonahhulmei* | KUMCC 21-0816^T^ | NR_182586 | NG_149052 | NG_148907 | ON009131 | ON009135 |
| *Thyridariella mangrovei* | PUFD98 | MG020434 | MG020437 | MG020440 | MG020443 | MG020445 |
| *Torula herbarum* | CBS 111855^T^ | KF443409 | KF443386 | KF443391 | KF443403 | KF443396 |
| *Torula hollandica* | CBS 220.69 ^T^ | KF443406 | KF443384 | KF443389 | KF443401 | KF443393 |
| *Xenoroussoella triseptata* | MFLUCC 17-1438^T^ | MT214343 | MT214437 | MT214391 | MT235767 | MT235804 |
